# Individualised prediction of longitudinal change in multimodal brain imaging

**DOI:** 10.1162/imag_a_00215

**Published:** 2024-07-03

**Authors:** Weikang Gong, Christian F. Beckmann, Stephen M. Smith

**Affiliations:** School of Data Science, Fudan University, Shanghai, China; Centre for Functional MRI of the Brain (FMRIB), Nuffield Department of Clinical Neurosciences, Wellcome Centre for Integrative Neuroimaging, University of Oxford, Oxford, United Kingdom; Radboud University Medical Centre, Department of Cognitive Neuroscience, Nijmegen, Netherlands; Donders Institute for Brain, Cognition and Behaviour, Radboud University Nijmegen, Nijmegen, Netherlands

**Keywords:** longitudinal changes, individualised prediction, multimodal brain images, deep learning

## Abstract

It remains largely unknown whether individualised longitudinal changes of brain imaging features can be predicted based only on the baseline brain images. This would be of great value, for example, for longitudinal data imputation, longitudinal brain-behaviour associations, and early prediction of brain-related diseases. We explore this possibility using longitudinal data of multiple modalities from UK Biobank brain imaging, with around 3,500 subjects. As baseline and follow-up images are generally similar in the case of short follow-up time intervals (e.g., 2 years), a simple copy of the baseline image may have a very good prediction performance. Therefore, for the first time, we propose a new mathematical framework for guiding the longitudinal prediction of brain images, providing answers to fundamental questions: (1) what is a suitable definition of longitudinal change; (2) how to detect the existence of changes; (3) what is the “null” prediction performance; and (4) can we distinguish longitudinal change prediction from simple data denoising. Building on these, we designed a deep U-Net based model for predicting longitudinal changes in multimodal brain images. Our results show that the proposed model can predict to a modest degree individualised longitudinal changes in almost all modalities, and outperforms other potential models. Furthermore, compared with the true longitudinal changes computed from real data, the predicted longitudinal changes have a similar or even improved accuracy in predicting subjects’ non-imaging phenotypes, and have a high between-subject discriminability. Our study contributes a new theoretical framework for longitudinal brain imaging studies, and our results show the potential for longitudinal data imputation, along with highlighting several caveats when performing longitudinal data analysis.

## 1 Introduction

The brain’s structure and function gradually change over time. These changes are reflected in (and potentially can then be predicted by) behavioural, clinical, cognitive, and demographic measures (referred to here as non-imaging derived phenotypes or nIDPs). Accurately detecting these brain changes can not only help the early design of personalised treatments for brain disorders but also bring new neuroscientific insights into brain-behaviour relationships (Douaud et al., 2022; Mills & Tamnes, 2014) and, from a practical perspective, may even allow for longitudinal data imputation. However, longitudinal studies of the brain are more difficult to conduct than cross-sectional studies, because of the need for rescanning subjects’ brain image data, which are expensive and difficult to acquire. Therefore, typically we need to use subjects with both baseline and follow-up data to perform longitudinal analysis, which reduces the usable range of datasets and/or subjects (for example, to date, UK Biobank has released data from over 40,000 subjects’ first imaging scan, but only about 3,500 of these also having had a second scan). One possible way to solve this problem is to synthesise longitudinal brain image data based only on baseline images using machine-learning techniques, and then perform the longitudinal analysis based on the synthesised images. However, it is largely unknown whether the underlying longitudinal changes of the brain can be predicted.

Previous work has shown that, for some neuroimaging modalities, it is possible to do longitudinal prediction. For example, Da Silva et al. (2021), Gafuroğlu et al. (2018), and Xia et al. (2021) showed that future T1-weighted (T1w) brain images could be synthesised based on just the baseline T1w image. Rachmadi et al. (2020) showed that white matter hyperintensities’ (WMH) evolution 1 year later could be predicted by the baseline WMH observed in T2-fluid attenuated inversion recovery (T2-FLAIR) images. Hong et al. (2019) also reported that infant diffusion MRI data can be predicted longitudinally. Other studies also showed that the brain functional connectome can also be predicted from the baseline connectome (Ghribi et al., 2021; Nebli et al., 2020). However, these studies only focus on one or two modalities with a few subjects. In addition, these studies only focus on the prediction of follow-up images. Little attention is paid to predicting the temporal changes, which is a key quantity of interest in longitudinal studies. Therefore, a more comprehensive study is needed to explore the possibility of longitudinal brain image prediction, and a more general framework should broaden our understanding of brain development and ageing.

In this study, we seek to answer the following questions: *Is it possible to predict subjects’ follow-up brain imaging data and their longitudinal changes based only on their baseline brain images?* Given this central question, we proposed the following sub-questions: (1) What is a suitable definition of longitudinal change in brain imaging? (2) Can we detect the existence of longitudinal change given our observed baseline data? (3) As baseline and follow-up images are intrinsically similar when we have short follow-up time intervals, what is the “null” correlation between predicted images and true images? (4) Can we determine whether a trained model is making change prediction rather than simple data denoising? (5) Does our method outperform other state-of-the-art approaches? (6) Do the predicted maps capture change patterns that are related to subjects’ nIDPs? (7) Can we explain which parts of the brain (in the baseline image) are used by the model to predict the longitudinal changes?

We used UK Biobank brain imaging data to address these questions, from which we extracted 50 different modalities from subjects’ structural, functional, and diffusion magnetic resonance imaging (MRI) scans from over 3,500 subjects longitudinally, with one baseline and one (on average 2 years later) rescan available (Table 1). The term “modality” here is defined as the voxel-level feature maps generated from different brain imaging scans, such as the fractional anisotropy (FA) from dMRI and Voxel based morphometry (VBM) maps from the T1w MRI. First, we propose a mathematical framework that models the longitudinal changes as a nonlinear function of the baseline image. This forms the basis of why we were able to predict longitudinal changes. Building on this framework together with an empirical estimation of data test-retest reproducibility, we show that we can (1) detect whether there exist temporal changes in the imaging data; (2) derive empirical baseline correlation of temporal change prediction; and (3) test whether a model is achieving change prediction or simply denoises the data. We then proposed a deep learning-based model, based on 3D U-Net (Çiçek et al., 2016), to perform longitudinal prediction, and a framework for explaining the deep prediction model. The motivation for using a U-Net based structure is that U-Net uses skip connections in corresponding feature maps with the same spatial resolutions in the downsampling and upsampling layers, which assist the model to focus on learning the changes in the follow-up images compared with the baseline.

**Table 1.** A description of 50 brain imaging modalities of UK Biobank dataset used in our study.

Abbreviation	Full description
rest k (k = 1-25)	Dual regression for IC k of 25 dimensional decomposition of rfMRI
task z1	z-statistics of emotion task contrast “shapes”
task z2	z-statistics of emotion task contrast “face”
task z5	z-statistics of emotion task contrast “faces>shapes”
task c1	Contrasts of parameter estimate (COPE) of emotion task contrast “shapes”
task c2	Contrasts of parameter estimate (COPE) of emotion task contrast “face”
task c5	Contrasts of parameter estimate (COPE) of emotion task contrast “faces>shapes”
TBSS-FA	Tract-Based Spatial Statistics - fractional anisotropy
TBSS-MD	Tract-Based Spatial Statistics - mean diffusivity
TBSS-MO	Tract-Based Spatial Statistics - tensor mode
TBSS-L1	Tract-Based Spatial Statistics - amount of diffusion along the principal directions 1
TBSS-L2	Tract-Based Spatial Statistics - amount of diffusion along the principal directions 2
TBSS-L3	Tract-Based Spatial Statistics - amount of diffusion along the principal directions 3
TBSS-OD	Tract-Based Spatial Statistics - orientation dispersion index
TBSS-ICVF	Tract-Based Spatial Statistics - intra-cellular volume fraction
TBSS-ISOVF	Tract-Based Spatial Statistics - isotropic or free water volume fraction
tracts	Summed tractography map of 27 tracts from AutoPtx in FSL
VBM	Voxel-based morphometry
Jacobian	Jacobian map of nonlinear registration of T1 image to MNI152 standard space
swMRI	T2* image derived from swMRI
T2 lesion	White matter hyperintensity map estimated by BIANCA
T1 nonlinear	T1 weighted image nonlinearly registered to MNI152 standard space
T2 FLAIR	T2 FLAIR image nonlinearly registered to MNI152 standard space
T1 warp (x,y,z)	The nonlinear warp field of T1 image registered to MNI152 standard space

We show that the proposed U-Net model is generic and easy to train. It can be configured to either predict each modality’s follow-up scan, or the change between follow-up and baseline scans, from the baseline scans (including the baseline scans from other modalities). It outperforms other state-of-the-art linear prediction models, such as Supervised BigFLICA (Gong et al., 2022), and other potential nonlinear approaches including Variational Autoencoder (VAE) (Kingma & Welling, 2013), other variants of U-Nets (e.g., Attention U-Net (Oktay et al., 2019), U-Net++ (Z. Zhou et al., 2018) and Nonlocal U-Net (Z. Wang et al., 2020)), and variants of generative adversarial networks (GANs) (e.g., Conditional GAN (Isola et al., 2017)). This is in agreement with the conclusion in a recent paper on the performance of U-Net in biomedical imaging tasks (Isensee et al., 2021). We evaluate the model’s performance by showing that it can meaningfully predict longitudinal changes in almost all 50 modalities in the UK Biobank, with the follow-up time interval being about 2 years. The prediction correlations are significantly larger than the chance level. We quantitatively and qualitatively show that our model can predict known change patterns in brain images, such as increased brain ventricles’ sizes observed in T1-weighted imaging-derived modalities. The predicted longitudinal changes have a similar or even improved out-of-sample predictive power for nIDPs, and can serve as a brain fingerprint of a subject (Finn et al., 2015). Therefore, our prediction model can aim to impute imaging data of missing subjects in longitudinal studies and perform early prediction of changes in brain imaging biomarkers.

## 2 Methods

In this section, we will introduce our three main contributions. In the first part, we start by introducing a general mathematical framework for guiding longitudinal prediction. In the second part, we show the details of the proposed deep U-Net model for predicting longitudinal brain imaging data. In the last part, we propose a method to “explain” the prediction model (i.e., generate a map of which parts of the brain are used to drive the prediction).

### 2.1 A general mathematical framework for longitudinal brain images

We are interested in whether the predicted image reflects longitudinal individual variability. However, as the correlation between input and output image is typically very high, simply copying (or copying and denoising) the baseline image may appear to have a high prediction performance. To address this problem, in this section, we propose a mathematical model to derive the “null” correlation of prediction, a model to judge the existence of temporal change, and a model to distinguish change prediction from data denoising.

We first set up the notations for our mathematical framework. Let the baseline image be A and its follow-up image B. We assume both A and B have been vectorised to size v×1 vectors, where v is the number of voxels. Non-brain voxels are removed. The model for baseline and follow-up images is:

$$\begin{array}{ccc} & A=A_{0}+E_{A} & \\ & B=B_{0}+E_{B} & \end{array}$$ (1)

where $A_{0}$ and $B_{0}$ are true underlying signals for the baseline and follow-up images, and $E_{A}∼N(0,\sigma^{2})$ and $E_{B}∼$ $N(0,\sigma^{2})$ are their noise terms. We assume baseline and follow-up images have the same level of noise.

The reason why we would expect we can use the baseline image A to predict the follow-up image B is that the true temporal change, Δ, might be a function of $A_{0}$:

$$\begin{array}{c} B_{0}=A_{0}+\Delta \\ \;\;\;\;\;\;\;\;\;\;\;\Delta =WA_{0}+f(A_{0}) \end{array}$$ (2)

where W is a linear transformation matrix (size v×v ) applied to $A_{0}$, and $f(A_{0})$ is a nonlinear function of $A_{0}$. We decompose the change into linear and nonlinear ones, which will be useful later. Common linear decomposition methods can be seen as learning W. For example, if we apply a partial least-squares regression to predict Δ using A, the product of coefficients of dimension reduction parts and prediction parts will approximate W. We further assume that the variance of $A_{0}$ is 1, and the mean of $A_{0}$ and $f(A_{0})$ is 0. This means that the brain image will change based on the pattern of $A_{0}$ (and possibly some patterns unrelated to $A_{0}$ which can be put into $f(A_{0})$ ). We propose to use the Pearson correlation, $r(x,y)=\text{cov(}x,y\text{)}\;/\sigma_{x}\sigma_{y}$, as the evaluation metric, where cov is the covariance and σ is the standard deviation. It is a commonly used metric for measuring spatial similarity for multiple structural and functional brain imaging modalities (e.g., Markello et al., 2022; Zheng et al., 2022), and it is a normalised metric which is readily interpretable for cross-modality comparison.

#### 2.1.1 Estimating the “null” prediction correlation of predicting longitudinal change from the baseline

The temporal difference B−A is the most intuitive definition for longitudinal change, and our task is to use A to predict it. As in a binary classification task, a null prediction correlation is needed for evaluating the model performance. When predicting B−A from A, a model might simply predict B−A as −A , as this appear to achieves high prediction correlation. This is because, in the null case of no temporal change, that is, $\Delta =B_{0}-A_{0}=0$, B−A becomes the difference of the noise terms, that is, $E_{B}-E_{A}$. Both B−A and A are usually dominated by the noise term $E_{A}$, resulting in high prediction correlation. More details can be seen from the mathematical formulas below.

Based on Eq. 2, the Pearson correlation coefficient between the temporal difference B−A and baseline A is:

$$\begin{array}{l} \begin{array}{cc} r\left(B-A,A\right)= & r\left(WA_{0}+f\left(A_{0}\right)+E_{B}-E_{A},A_{0}+E_{A}\right) \end{array}\\ \;\;\;\;\;\;\;\;\;\;\;\;\;\;\;\;\;\;\;\;\;\;\;\;\;\;\;\;\;\;\;\approx \frac{\text{cov}\left(WA_{0},A_{0}\right)-\sigma^{2}}{\sqrt{\left(\text{var}\left(WA_{0}\right)+\text{var}\left(f\left(A_{0}\right)\right)+2\sigma^{2}\right)\left(1+\sigma^{2}\right)}} \end{array}$$ (3)

where we have used the fact that the nonlinear function of $A_{0}$ is not linearly correlated with $A_{0}$, that is, $\text{cov(}f(A_{0}),$ $A_{0}\text{)}=0$ , and noise terms in A and B are not correlated, that is, $\text{cov(}E_{A},E_{B}\text{)}=0$ , and terms with $A_{0}$ are not correlated with noise terms, for example, $\text{cov(}E_{B},A_{0}\text{)}=0$ .

If there are no true temporal changes (Δ=0 , i.e., the null scenario), the above correlation reduces to:

$$\begin{array}{cc} r\left(B-A,A\right) & =r\left(E_{B}-E_{A},A_{0}+E_{A}\right)=-\frac{1}{\sqrt{2+2/\sigma^{2}}} \end{array}$$ (4)

which will vary within the interval $\left(-\frac{1}{\sqrt{2}},0\right)$, with σ∈(0,+∞) .

To estimate the null-case r(B−A,A) , we can estimate σ, that is, the noise level in baseline or follow-up images. An example for resting-state dual-regression maps is shown in the Supplemental Material. More importantly, an extension of the above analysis is to derive the null prediction correlation of predicting B from A, that is, predicting the follow-up image from the baseline. When there are no (true underlying) temporal changes, that is, $\Delta =B_{0}-A_{0}=0$ , both A and B contains the $A_{0}$ term, so predicting B as A is the null prediction correlation.

#### 2.1.2 A model for testing the existence of longitudinal change

We then propose another question: can we judge whether temporal change exists? To do this, we rely on the difference between the observed baseline-follow-up correlation and the “null” correlation.

For example, in the above section, we showed that the “null” correlation between B−A and A might be -0.49 for the default mode network. However, we can estimate, from real data, the r(B−A,A)=−0.63 . The difference between observed and “null” correlation means that there may exist some temporal change in this modality, that is, Δ≠0 .

In more detail, from Eq. 3, we can see that when Δ≠0 , the term $\text{cov(}WA_{0},A_{0}\text{)}$ influences the observed temporal correlation. When it is smaller than 0, the observed r(B−A,A) can be smaller than the “null” case correlation. This means that in the population, the linear part of the temporal changes, that is, $WA_{0}$, should be negatively correlated with $A_{0}$.

A similar result can be derived from the correlation between baseline and follow-up images:

$$\begin{array}{l} \begin{array}{cc} r\left(B,A\right) & =r\left(A_{0}+WA_{0}+f\left(A_{0}\right)+E_{B},A_{0}+E_{A}\right) \end{array}\\ \;\;\;\;\;\;\;\;\;\;\;\;\;\;\;\;\;\;\;\;\;\;\;\;\;\approx \frac{\text{cov}\left(\left(I+W\right)A_{0},A_{0}\right)}{\sqrt{\left(\text{var}\left(\left(I+W\right)A_{0}\right)+\text{var}\left(f\left(A_{0}\right)\right)+\sigma^{2}\right)\left(1+\sigma^{2}\right)}} \end{array}$$ (5)

When Δ=0 , this correlation reduces to the data reproducibility. This is because $r(B,A)=r(A_{0}+E_{B},A_{0}+E_{A})$ .

For example, in our UKB data, we estimated that the data reproducibility for the default mode network (rest 1) is 0.34 by split-half data (which should be higher if we can use the full data). At the same time, the observed r(B,A) is only 0.23 (as shown later in Fig. 4). When $\text{cov(}WA_{0},A_{0}\text{)}<0$ and/or there are nonlinear changes (which influences the variance term), the r(B,A) can be smaller than the data reproducibility.

#### 2.1.3 Distinguishing change prediction and data denoising

Another under recognised issue with longitudinal prediction is that we get very good predictions if the trained model just denoises the baseline image. This is because when a model can decrease the noise variance of the baseline image, the Pearson correlation between the true and predicted images can be increased. Therefore, it is critical to be able to define methods that distinguish between whether the model is doing change prediction versus data denoising.

Suppose our prediction model is linearly decomposed into change prediction and data denoising parts, that is, $g(\;\cdot \;)=g_{1}(\;\cdot \;)+g_{2}(\;\cdot \;)$ , where $g_{1}(\;\cdot \;)$ does change prediction and $g_{2}(\;\cdot \;)$ does denoising. Then, the following equations show the correlation between predicted follow-up images, PredB, and the true follow-up image, B:

$$\begin{array}{l} r(\text{PredB,B})=r(g(A),B)\\ \;\;\;\;\;\;\;\;\;\;\;\;\;\;\;\;\;\;\;\;\;\;\;\;\;\;\;=r(g_{1}(A_{0})+g_{2}(E_{A}),(1+W)A_{0}+f(A_{0})+E_{B}). \end{array}$$ (6)

If $g_{1}(\;\cdot \;)$ learns nothing but is simply a scaled copy of $A_{0}$ (no change prediction, $g_{1}(A_{0})=A_{0}$) and only $g_{2}(\;\cdot \;)$ is active (pure denoising), the correlation between the predicted follow-up image and the true follow-up image is:

$$\begin{array}{l} r(\mathrm{Pr}edB,B)=r(A_{0}+g_{2}(E_{A}),(I+W)A_{0}+f(A_{0})+E_{B})\\ \;\;\;\;\;\;\;\;\;\;\;\;\;\;\;\;\;\;\;\;\;\;\;\;\;=\frac{\mathrm{cov}(A_{0},(I+W)A_{0})}{\sqrt{\left[1+\mathrm{var}(g_{2}(E_{A})][\mathrm{var}((I+W)A_{0})+\mathrm{var}(f(A_{0}))+\sigma^{2}\right]}}\\ \;\;\;\;\;\;\;\;\;\;\;\;\;\;\;\;\;\;\;\;\;\;\;\;\;=\frac{1}{\sqrt{1+\mathrm{var}(g_{2}(E_{A})}}\frac{\mathrm{cov}(A_{0}B_{0})}{std(B)} \end{array}$$ (7)

By comparing Eq. 7 with r(B,A) (Eq. 5), we can see that the only difference is that the $\sigma^{2}$ term becomes $\text{var}\;\text{(}\;g_{2}(E_{A})$ . The latter is smaller when the model denoises the data, so it is possible that we can still get a non-null correlation.

Intuitively, if a model g only does data denoising, feeding the follow-up image B into this model and estimating the correlation with the baseline image A should be similar to feeding the baseline image A and estimating the correlation with B. This is because if g only learns denoising, there should be no influence on whether we denoise the baseline or follow-up data on the estimated correlation, that is, decreasing $E_{A}$, ($g_{2}(E_{A})$ ), should be similar to decreasing $E_{B}$, ($g_{2}(E_{B})$ ), in Eq. 7. We can see this more clearly by proposing the following model. The model is based on an assumption that the pattern of change between 2 years is similar, that is, if C is the 4-years follow-up image, then the difference between A and B should be similar to B and C. Therefore, we can derive the correlation between C and B as:

$$\begin{array}{l} C=C_{0}+E_{C}=B_{0}+\Delta +E_{C}\\ \Delta =WB_{0}+f\left(B_{0}\right)\\ r\left(C,B\right)\approx r\left(B,A\right)=r\left(B,C-2\Delta \right)\to \frac{\text{cov}\left(B_{0}C\right)}{\text{std}\left(C\right)}\approx \frac{\text{cov}\left(B_{0}\left(C-2\Delta \right)\right)}{\text{std}\left(C-2\Delta \right)} \end{array}$$ (8)

where r(C,B)≈r(B,A) is our assumption.

Then, if we feed B into our trained model g( · ) (suppose the output is PredA), if g( · ) only denoises the data, we have:

$$\begin{array}{l} r(predA,A)=r(g(B),A)\\ \;\;\;\;\;\;\;\;\;\;\;\;\;\;\;\;\;\;\;\;\;\;\;\;\;\;=r(g_{1}(B_{0})+g_{2}(E_{B}),C-2\Delta )\\ \;\;\;\;\;\;\;\;\;\;\;\;\;\;\;\;\;\;\;\;\;\;\;\;\;\;=r(B_{0}+g_{2}(E_{B}),C-2\Delta ) \end{array}$$

$$\begin{array}{l} \;\;\;\;\;\;\;\;\;\;\;\;\;\;\;\;\;\;\approx \frac{1}{\sqrt{1+\text{var}\left(g_{2}\left(E_{B}\right)\right)}}\frac{\text{cov}\left(B_{0}\left(C-2\Delta \right)\right)}{\text{std}\left(C-2\Delta \right)}\\ \;\;\;\;\;\;\;\;\;\;\;\;\;\;\;\;\;\;\approx \frac{1}{\sqrt{1+\text{var}\left(g_{2}\left(E_{B}\right)\right)}}\frac{\text{cov}\left(B_{0}C\right)}{\text{std}\left(C\right)}\\ \;\;\;\;\;\;\;\;\;\;\;\;\;\;\;\;\;\;\approx \frac{1}{\sqrt{1+\text{var}\left(g_{2}\left(E_{B}\right)\right)}}\frac{\text{cov}\left(A_{0}B\right)}{\text{std}\left(B\right)}\\ \;\;\;\;\;\;\;\;\;\;\;\;\;\;\;\;\;\;\approx r\left(\text{PredB},B\right) \end{array}$$ (9)

Then, if g(B) is closer to C than A, which means g is doing change prediction, we have r(PredB,B)>r (PredA,A).

This now means that we can distinguish between g that only does denoising or do longitudinal prediction by directly comparing r(PredB,B) and r(PredA,A).

#### 2.1.4 Residualisation of baseline and follow-up images

A practical problem when predicting B using A is that the model may be significantly biased towards learning to predict the population average of B. This is because, for all modalities, there is a population average pattern that can be highly similar to each subject’s image. For example, in task contrast maps, every subject often tends to have activations in similar brain areas. The population average pattern can dominate each subject’s image so that a model might find that simply predicting the population mean can achieve a low mean square error loss. There are two ways to eliminate this population average effect, both of which have shown success in predicting task activation maps using resting-state fMRI data. The first is to use contrastive loss (Ngo et al., 2021), which is a loss function that simultaneously minimises both the prediction error and the similarity among the predicted maps of different subjects. The second approach is residualisation, which regresses the population average map out of each of the individual images using linear regression, and creates individualised predictions based on the residualised maps (Zheng et al., 2022). The first approach requires optimising a balancing parameter between two losses, which is computationally inefficient. We therefore used the second approach to enforce learning parameters that characterise longitudinal change at an individual level.

In detail, we will denote the residualised (with respect to the population average) baseline image as ResA, and the residualised follow-up image as ResB. Let $\bar{A}$ and $\bar{B}$ be the population average images in the training set, then each subject’s ResA and ResB are obtained as:

$$\begin{array}{ccc} & \;\;\;\;\text{A}=\bar{A}\hat{\beta }+\text{ResA} & \\ & \text{ResA}=A-\bar{A}\hat{\beta } & \\ & \text{ResB}=B-\bar{A}\hat{\beta } & \end{array}$$ (10)

Note that here A and B are vectorised and non-brain voxels are removed before being fed into the linear regression. $\hat{\beta }$ is the least-square estimate of regression coefficients when regressing $\bar{A}$ out of A. Then, this $\hat{\beta }$ is applied to estimate ResA and ResB. We can also use the coefficient of regressing out $\bar{B}$ from B to generate ResB, but we find there is little difference in practice. Furthermore, one added advantage of using $\bar{A}$ only is that we can reconstruct the estimated original B from the predicted ResB without knowing the true original B.

#### 2.1.5 Definitions of longitudinal changes

The above sections explain why the success of predicting follow-up images does not necessarily mean we can accurately capture the longitudinal change patterns. In addition, in longitudinal analysis, we are often interested in how the temporal changes in imaging features relate to nIDPs (non-imaging-derived phenotypes, i.e., other non-imaging variables such as cognitive scores or disease outcomes) or changes in nIDPs. However, simply defining *temporal difference* as ResB-ResA (or B−A ) has a non-zero null prediction correlation, as shown above (section 2.1). We need to estimate a null prediction correlation for this metric as a reference for model prediction.

To find a metric that has zero null-case correlation, we propose to define a *residual temporal difference* metric as ResB\ResA, that is, regressing out ResA from ResB:

$$\begin{array}{ccc} & \text{ResB}=\text{ResA}\times \hat{\beta } & \\ & \text{ResB}\backslash \text{ResA}=\text{ResB}-\text{ResA}\hat{\beta } & \end{array}$$ (11)

where $\hat{\beta }$ is the least-squares estimate of the coefficients when regressing ResA out of ResB. Using this definition, ResA has zero correlation with ResB\ResA, so that in the null case of no temporal changes, the correlation between ResB\ResA and ResA is exactly zero. The intuitive interpretation of ResB\ResA is that the information in ResB can not be linearly represented by ResA. Therefore, if we choose this metric, we aim to examine the prediction performance of the nonlinear part of the changes, that is, $f\left(A_{0}\right)$. This is because, in the regression, we not only regress out the original ResA in the baseline data but also regress out the linear part of temporal changes. A further concern is whether we can accurately remove all linear parts related to $A_{0}$. A concern with ResB\ResA may arise due to the regression dilution problem: when ResA is a noisy modality, ResB\ResA may still contain information in $A_{0}$ because of the underestimation of regression coefficients.

### 2.2 A deep U-Net model for longitudinal prediction

**Fig. 1.**
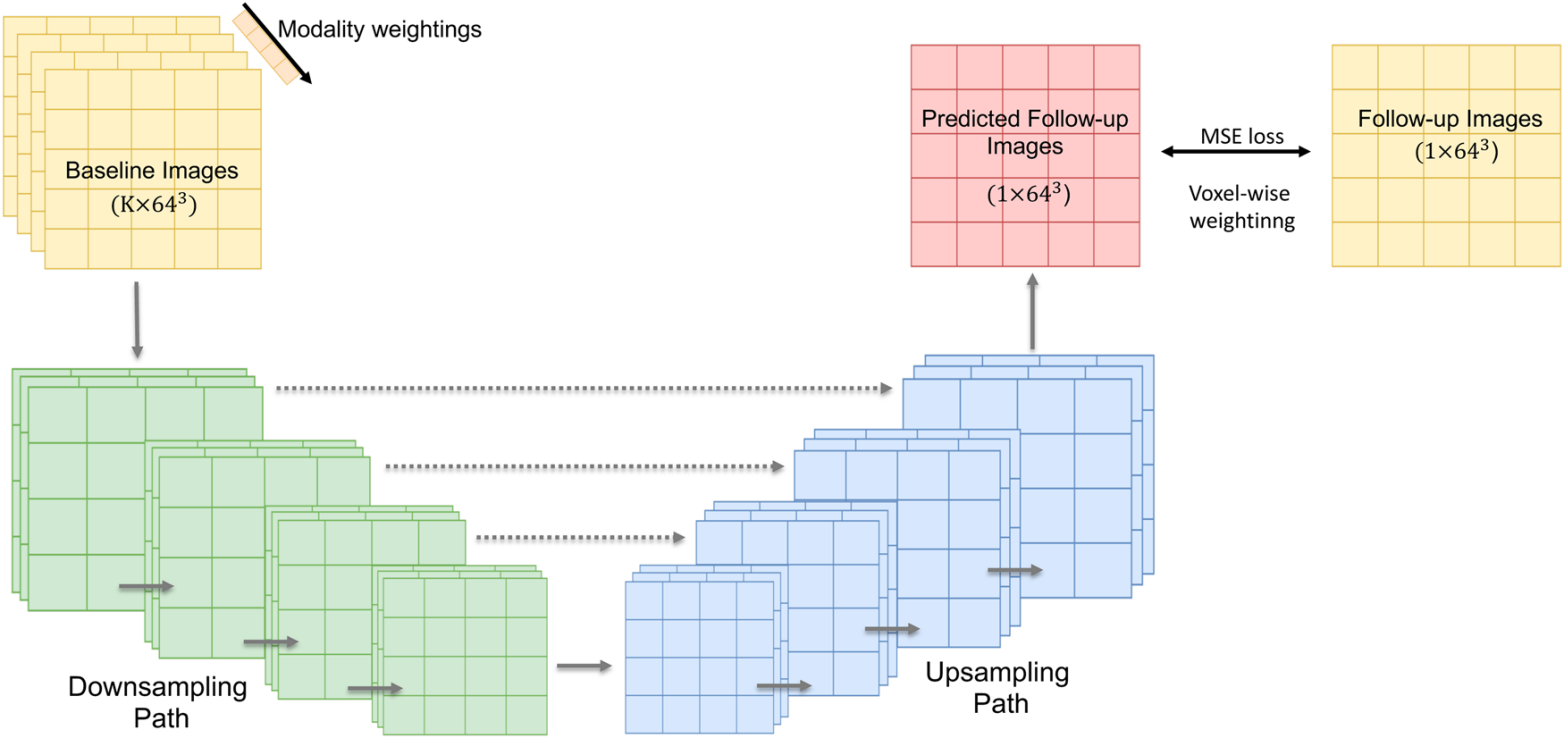
An overview of the proposed U-Net structure for longitudinal brain image prediction. The network has four downsampling and upsampling layers with skip connections. The numbers $a\times b^{3}$ represent the number of channels (a) and spatial voxel dimensions (b) of the inputs and outputs.

U-Net is a popular deep neural network originally designed for image segmentation. We adopt a U-Net structure here with a modified voxel-wise mean square error loss to perform longitudinal prediction. Figure 1 shows the network architecture. The input of the network is a baseline image, and the output is the predicted follow-up image. The network has a downsampling and an upsampling path, each with four layers. In the downsampling path, each layer contains two 3×3×3 convolutions followed by batch normalisation and a rectified linear unit (RELU), and followed by a 3×3×3 average pooling layer with strides of 2 in each dimension. In the upsampling path, each layer contains a 3×3×3 upconvolution, followed by two 3×3×3 convolutions and a batch normalisation and an RELU. Skip connections are added between feature maps in the downsampling and the upsampling path with the same spatial resolution, which is one of the key quantities that differs from Autoencoder-based models. The number of channels doubles in each layer of the downsampling path while becoming halved in each layer of the upsampling path. In the final layer, a 1×1×1 convolution reduces the number of channels to the target output.

When we use multiple (K) baseline modalities to do longitudinal prediction, the baseline images are simply concatenated, forming a four-dimensional input to the U-Net. Therefore, the number of channels in the first layer of the U-Net becomes K. We also add a positive weight vector to the network to learn the contributions of different modalities to the prediction. The length of the vector equals K, and each of the elements is multiplied by one modality map before the first U-Net downsampling. Therefore, after network training, modalities with higher contributions to the prediction of follow-up images should have higher weights.

We used mean square error at each voxel as the loss function, because our target image is a continuous one. The losses are only calculated in voxels inside the brain. To combine losses across multiple voxels, we use a weighted sum of voxel-wise mean square error. The weights of voxels are learned based on their prediction uncertainty (Kendall et al., 2018). Specifically, let the loss function at voxel i be $l_{i}$, then the overall loss is:

$$L=\sum_{i=1}^{v}\left(\frac{l_{i}}{2\sigma_{i}^{2}}+\text{log}\left(\sigma_{i}+1\right)\right)$$ (12)

where $\sigma_{i}>0$ is a learnable parameter. This idea was originally developed for multi-task learning (Kendall et al., 2018). Here, we treat the prediction at each voxel as a different “task”. The weights among voxels are proportional to the inverse of the residual prediction variance. This is analogous to a Bayesian linear regression with unknown residual variance (Bishop, 2006). The motivation is that the tasks with larger error/uncertainty will be given lower weights (Kendall et al., 2018). The performance is improved compared with using MSE loss without voxel-wise weighting empirically, and the learning of weighting parameters is efficient.

### 2.3 Explaining the longitudinal prediction model

After generating the predicted image, a natural follow-up question is: which part of the brain in the baseline image contributes to the prediction of longitudinal changes? Existing methods for explaining the “black-box” deep model mainly focus on classification or regression tasks, and include saliency maps (Simonyan et al., 2013) and Class Activation Maps (CAM) (B. Zhou et al., 2016). Unlike classification and regression tasks, the output of U-net based modelling is a spatial map. Therefore, we propose a weighted saliency maps approach to explain the longitudinal prediction model.

The goal of model explanation is, for a given subject, to generate an image which represents the importance of each input voxel in predicting the corresponding follow-up output image. Our approach has two steps. In the first step, we adopt the image-specific saliency visualisation technique from (B. Zhou et al., 2016) to visualise the contribution of each voxel. That is, for a given input A and model output B, we compute the derivative of B at voxel i ($B_{i}$) with respect to the image A: $E_{i}=\partial B_{i}/\partial A$ . The magnitude of this derivative image $E_{i}$ reflects which voxel needs to be changed the least to affect the prediction score the most. In the second step, we combine the $E_{i}$ maps across multiple output voxels to get a single explanation map. To this end, we first threshold the predicted image to get its top k voxels with the largest absolute scores, and then take the sum of these voxels’ absolute saliency maps, $E=\sum E_{i}$, to get the explanation for this subject. In this work, we set the k as the 5% voxels with the highest absolute values in the predicted image. We also estimated the population mean explanation map as the average of all individual explanation maps.

### 2.4 Experiments

#### 2.4.1 Comparing with other approaches

There are broad classes of models that can perform longitudinal prediction. We choose a recent method called SuperBigFLICA (Gong et al., 2022) as a baseline linear method owing to its state-of-the-art performance on multimodal brain imaging data. In addition, the Variational Autoencoder (VAE) (Kingma & Welling, 2013) is a Bayesian generative model widely used for image generation and denoising. Similarly, another comparison is against conditional generative adversarial networks (cGANs), which use adversarial losses for image synthesis (Isola et al., 2017). We also compared the proposed U-Net structure with more advanced variants of U-Net, that is, Attention U-Net (Oktay et al., 2019), U-Net++ (Z. Zhou et al., 2018), and Nonlocal U-Net (Z. Wang et al., 2020).

In SuperBigFLICA, We added skip connections from the input directly to the output, and used the Adam optimiser (Kingma & Ba, 2014) for all learnable parameters (instead of using combined optimisers as done in the original work), which empirically improves the performance. For the VAE, we adopt the same structure as the proposed U-Net, with skip connections removed, while in cGANs, the structure of the generator network is the same as our proposed U-Net, and the weight between adversarial loss and voxel-wise MSE loss varies between 0 and 1. The same backbone structure as the proposed U-Net is used in the advanced U-Nets, with different attention modules added.

#### 2.4.2 Data

Voxel-wise neuroimaging data of 50 modalities of 3,572 subjects in the UK Biobank brain imaging dataset were used, including: (1) 25 “modalities” from the resting-state fMRI ICA dual-regression spatial maps (Miller et al., 2016); (2) 6 modalities from the emotion task fMRI experiment: 3 contrast (shapes, faces, faces>shapes) of Z-statistics and 3 *contrasts of parameter estimate* (COPE) maps (Miller et al., 2016) that reflect %BOLD signal change; (3) 10 diffusion MRI derived modalities (9 TBSS features, including FA, MD, MO, L1, L2, L3, OD, ICVF, ISOVF (Smith et al., 2006; Zhang et al., 2012) and a summed tractography map of 27 tracts from AutoPtx in FSL (De Groot et al., 2013)); (4) 3 T1-MRI derived modalities (standard space T1w image; grey matter volume; Jacobian deformation map (which shows expansion/contraction generated by the nonlinear warp to standard space, and hence reflects local volume) in the volumetric space; and the nonlinear warp maps (which reflect the shape changes in the nonlinear registration of an individual T1w image to the MNI152 standard space in each voxel in the x-, y-, and z-directions, so that it has 3 channels)); (5) 1 susceptibility-weighted MRI map (T2-star image); and (6) 2 T2-FLAIR MRI derived modality (standard space T2-FLAIR image; white matter hyperintensity map estimated by BIANCA (Griffanti et al., 2016)). The UK Biobank imaging data were mainly preprocessed using FSL (Jenkinson et al., 2012; Smith et al., 2004) following an optimised processing pipeline (Alfaro-Almagro et al., 2018) (https://www.fmrib.ox.ac.uk/ukbiobank/). A description of these 50 modalities is shown in Table 1.

These 3,572 subjects have both baseline and follow-up T1w images, and a small proportion of them have some other missing modalities. The follow-up images are scanned on average 2 years after the baseline images. We also used 15,697 nIDPs from UK Biobank (Alfaro-Almagro et al., 2018). We performed z-score normalization across subjects for each nIDP. We did not regress out confounding variables from brain imaging data or nIDP data. We exclude an nIDP if it has more than 1,000 missing values in these 3,572 subjects, or is constant in the training set (e.g., for many disease variables that have only a few subjects affected, it is possible that no disease affected subjects in the training set).

### 2.5 Implementation details

The U-Net model is implemented using the Pytorch framework. The input and output are first downsampled to 64×64×64 images. The intensity of the input image is then normalised to between zero and one by min-max normalisation before being fed into the model. The intensity of the output image is not normalised. We did not perform any data augmentation, because empirically, it does not improve model performance. We used the Adam optimizer (Kingma & Ba, 2014) for parameter optimization. The learning rate is set to 0.001 , and all other parameters in Adam are set as default. The mini-batch size is 16 subjects. The total number of epochs (number of times the full data passes through the model) is 40, and the learning rate decreases by a cosine rule. The model weights are initialised by Gaussian-distributed random numbers of mean 0 and variance 1.

A random subset of 2,500 subjects is used in training, while another random subset of 200 subjects is used as validation, and the remaining 852 subjects are used for testing and reporting the performance. The test set size is different for different modalities due to different missing data proportions. All evaluations are based on the same train/validation/test splitting. We used the mean Pearson correlation (r) and the mean absolute error (MAE) between the predicted and the true images as the evaluation criteria. Before calculating these two metrics, we excluded voxels outside the brain and vectorised the 3-dimensional image.

### 2.6 Evaluation of model prediction

Four complementary methods are proposed to evaluate the proposed model in different ways. The first is the most straightforward, which examines the model’s prediction performance by computing the correlation or MAE between the predicted image and true image, and comparing its value with the null case. This evaluation is applied in both follow-up images and residual temporal difference images.

The second method overcomes a potential limitation of the first one. In the first, we can still not distinguish whether the model can truly predict longitudinal changes or simply perform data denoising. We showed in section 2.1.3 that even if the deep model is simply denoising the data, we can potentially get a higher correlation value than the null case. We, therefore, propose a test to evaluate whether the model is doing change prediction: Given a trained deep network g(·), we feed the baseline image ResA and the follow-up image ResB into it separately, getting the outputs of the network as PredResB and PredResA (respectively). We then compare the correlation values r(ResB, PredResB) against r(ResA, PredResA). If the network is purely denoising, we show in section 2.1.3 that these two metrics should be very similar. On the contrary, if the former is larger than the latter, we show that the network can predict the longitudinal change. We further compare the above two correlations using the residual temporal differences maps, that is, r(ResB\ResA, PredResB\ResA) against r(ResA\ResB, PredResA\ResB).

The third method evaluates the discriminability of predicted maps based on ideas originally developed as the functional connectome fingerprint (Finn et al., 2015). A model can achieve individualised prediction if the predicted spatial maps can be a subject’s fingerprint, that is, the predicted maps correlate most to the subjects themselves but less so with other subjects. We tested whether the predicted maps could be identified based on their correlation with the true maps. We estimate the mean identification rate as the proportion of subjects whose within-subject correlation is the largest among all between-subject correlations. This evaluation is applied in both follow-up images and residual temporal difference images.

The fourth method compared the prediction performance of nIDPs between the predicted and the true residual temporal difference image. As our goal in brain image analysis is often linking image data to non-imaging variables, we want to test whether the predicted images have a similar or even improved nIDP prediction power compared with the original images. We investigated 15,697 nIDPs from UK Biobank, which include 17 groups of variable types, including early life factors, lifestyle factors, physical body measures, cognitive test scores, health outcomes, and mental health variables, previously used in C. Wang et al. (2022). These variables are measured at four time points, with two before the brain imaging scans, and one at the baseline brain imaging scans, and one at the follow-up scans. Specifically, for each modality, we calculated PredResB\ResA and ResB\ResA. Then, we used principal component analysis to reduce the residual temporal difference data into 500 orthogonal components, and then applied elastic-net regression, from the glmnet package (Zou & Hastie, 2005), to predict each of the nIDPs in UK Biobank. Elastic-net regression is widely used and has been shown to achieve robust and state-of-the-art performance in many neuroimaging studies (Cui & Gong, 2018; Jollans et al., 2019). We use the residual temporal difference image here because we want to eliminate all the effects of the baseline image on predicting nIDPs. In the simple temporal difference image, that is, ResB-ResA, we are not able to achieve this.

## 3 Results

### 3.1 Prediction performance of follow-up image

**Fig. 2.**
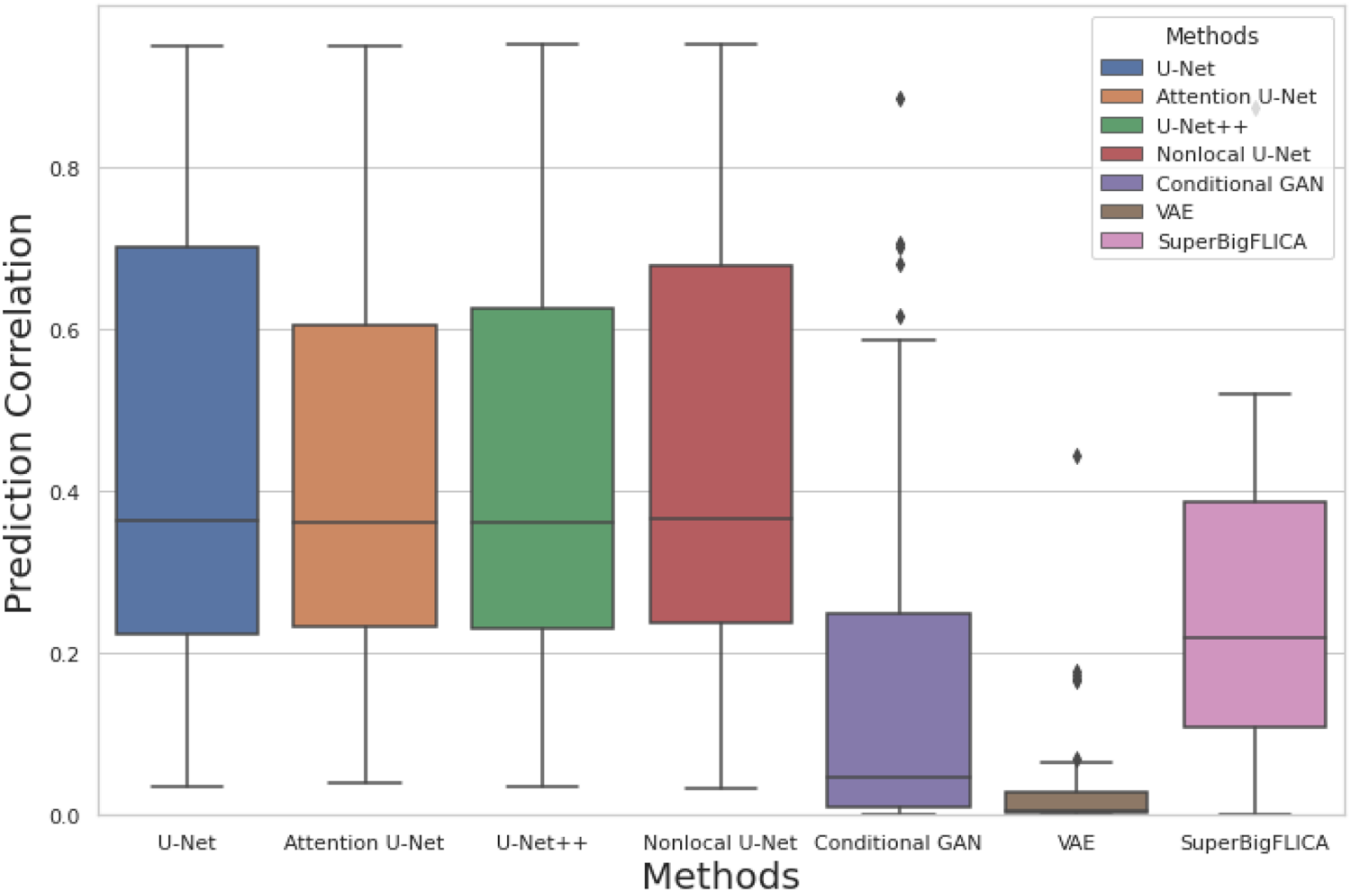
Comparison of prediction correlations of our method (U-Net) against other methods. Each boxplot shows the prediction correlation of the follow-up image using the baseline image with different methods across 50 brain imaging modalities in the UK Biobank dataset.

**Fig. 3.**
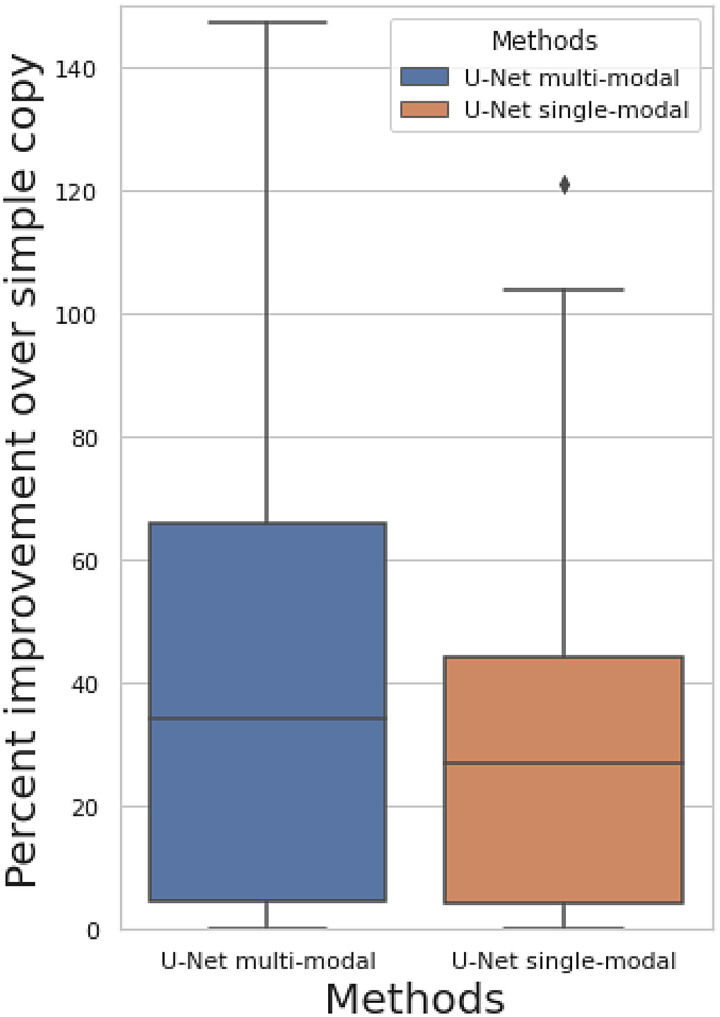
Percent improvement of predicting of follow-up image when comparing against simply copying the baseline image as the prediction among 50 brain imaging modalities.

Figure 2 shows a comparison of different approaches for longitudinal prediction across 50 brain imaging modalities in the UK Biobank datasets. Here, the input is the baseline image, and the target is the follow-up image of one specific modality. Both the baseline and follow-up images have been residualised. We can see that U-Net based approaches achieve the best performances. More advanced U-Nets do not show any significant advantages compared to our proposed U-Net. The U-Net based methods outperform simply copying the baseline image as the prediction of the follow-up image (Fig. 3). The other three methods, that is, VAE, cGANs, and SuperBigFLICA, perform significantly worse than the U-Net based approaches. Although successful in many computer vision tasks, the failure of VAE and cGANs here may be because they are not well suited to the noisy, high-dimensional, and small-sample size brain image data. The failure of SuperBigFLICA may be due to its linear nature so that important nonlinear information is lost. Note that Figure 2 does not demonstrate whether the methods are successful in estimating longitudinal change, as it only shows the extent to which the second scan is predicted (which might be just the aspects of the second scan that are similar to the first).

Figure 3 shows the percent improvement of prediction correlation of our approaches when comparing against simply copying the baseline image as the prediction (section 2.1.1). The prediction target is still the follow-up image. We show results for both the multimodal U-Net (all 50 modalities are inputs) and single-modal U-Net (only target modality is the input). Both models show non-null prediction performance, with a mean improvement of 41% for multimodal model and 27% for single-modal model. The prediction correlation for each modality is shown in Supplementary Figure 1. The multimodal model has a small performance improvement compared with the single-modal model, indicating that the complementary features in different modalities can be learned to assist longitudinal prediction, but the primary information contributing to the prediction exists in the same modality as the target. The fact that the U-Net outperforms simply copying the input image to the output does not necessarily demonstrate successful longitudinal (change) prediction, because the improvement might also be due to image denoising, or a combination of both.

**Fig. 4.**
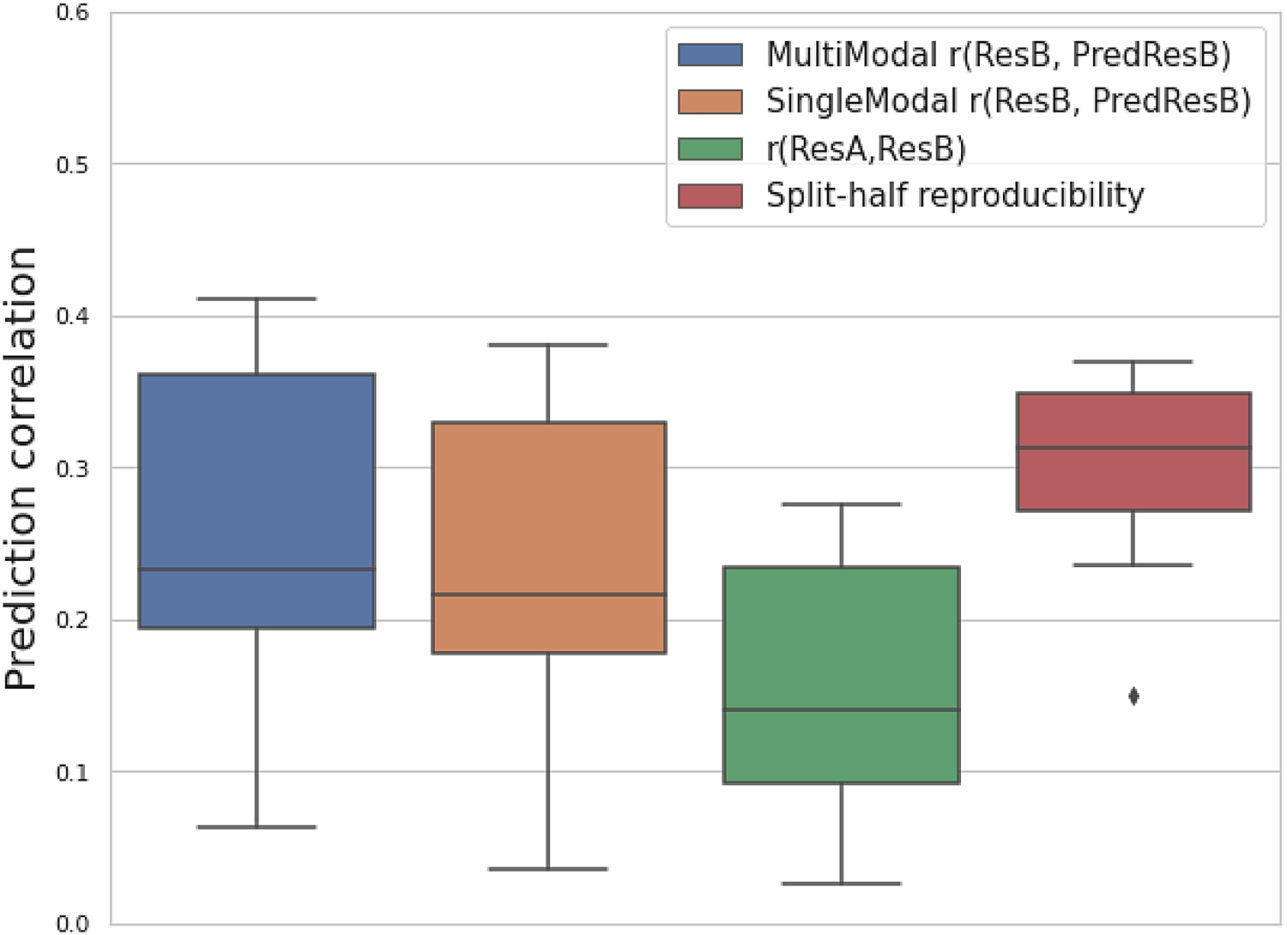
An analysis of model performance on predicting follow-up images based on the split-half reproducibility of resting-state fMRI modalities. For 25 resting-state fMRI modalities, the boxplots show the prediction correlation of follow-up images (ResB) with multimodal and single-modal U-Net (blue and orange); and a baseline of copying baseline image (ResA) as the prediction (green); and the test-retest reproducibility by splitting the time series in half (red).

Figure 4 compares the test-retest correlation of 25 resting-state fMRI modalities (estimated by splitting the time series of the follow-up data into two) with the prediction correlation of the follow-up image and with the observed correlation. As shown in section 2.1.2, a lower correlation between baseline and follow-up than the test-retest reproducibility indicates temporal change. The changes may be linearly and negatively related to the baseline and nonlinearly related to the baseline.

For the comparison between prediction correlation and test-retest reproducibility, theoretically, among 25 resting-state modalities, 8 of these have a larger prediction correlation than the test-retest reproducibility when using multimodal input, while 2 of them are larger when using single-modal input (Supplementary Fig. 2). As the input baseline image has a similar SNR to the follow-up image, we would expect a prediction correlation that is similar to the full-data test-retest reproducibility when the underlying temporal changes are perfectly predicted. Here, as we only used split-half data to estimate the reproducibility, the reproducibility should be lower than the full-data case. Therefore, we would expect most of the time, the prediction correlation of a single modality to be smaller than the full-data test-retest reproducibility. As a result, we can even observe that 23 of the 25 single modality prediction correlations are smaller than the split-half reproducibility. In multimodal prediction, we observe 8 modalities that can exceed the split-half reproducibility, indicating clear advantages of integrating complementary information across different modalities.

### 3.2 Prediction performance of longitudinal changes

**Fig. 5.**
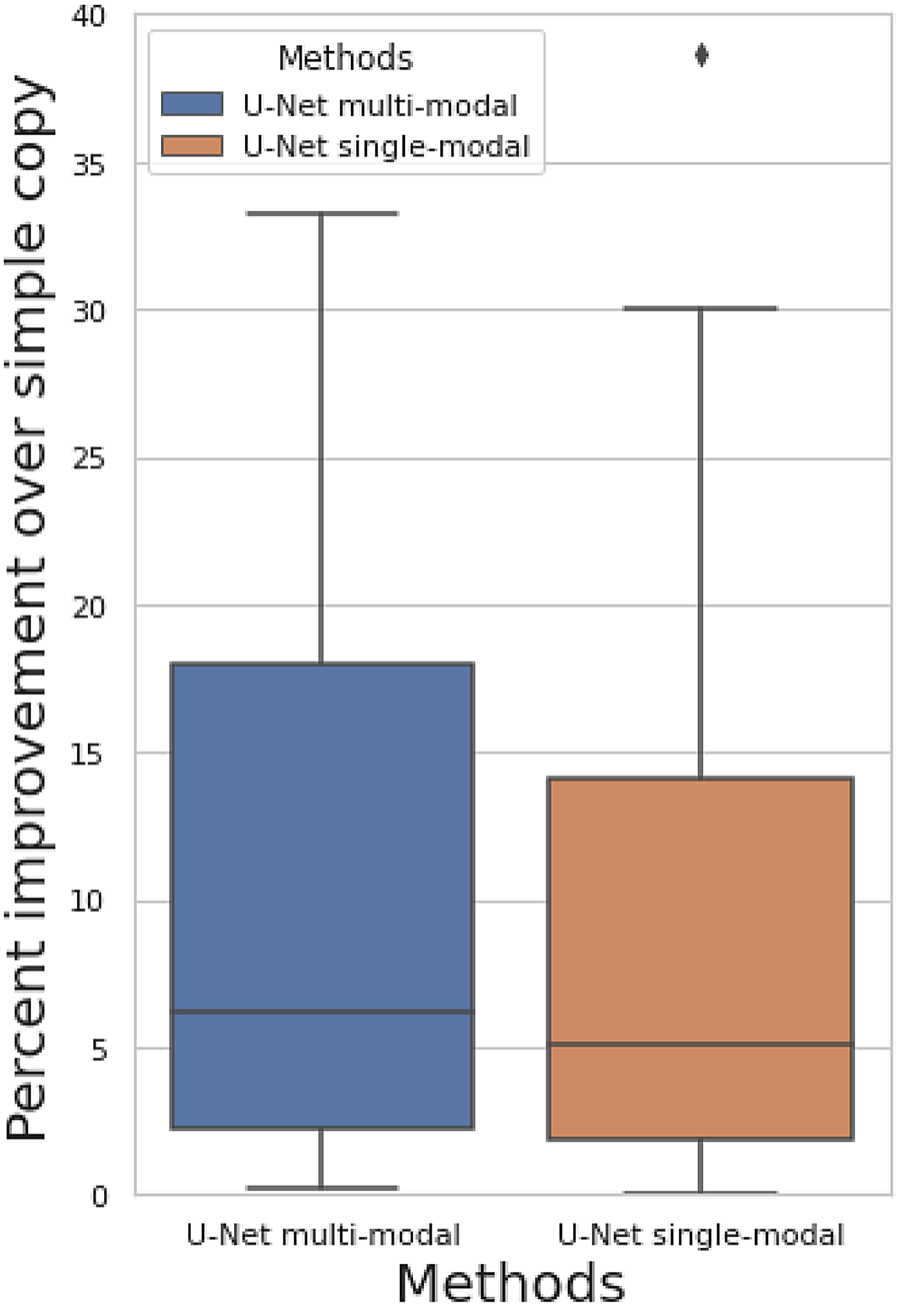
Percent improvement of predicting of temporal difference image (ResB-ResA) when comparing against simply copying the negative baseline image (-ResA) as the prediction among 50 brain imaging modalities.

Figure 5 shows the percent improvement of predicting of temporal difference image (ResB-ResA) when comparing against simply copying the negative baseline image (-ResA) as the prediction among 50 brain imaging modalities. Across all modalities, the relative improvement than the null is smaller than predicting the follow-up image directly. The accuracy of single modality prediction was lower by only a small amount than multimodal prediction. The prediction correlation for each modality is shown in Supplementary Figure 3.

**Fig. 6.**
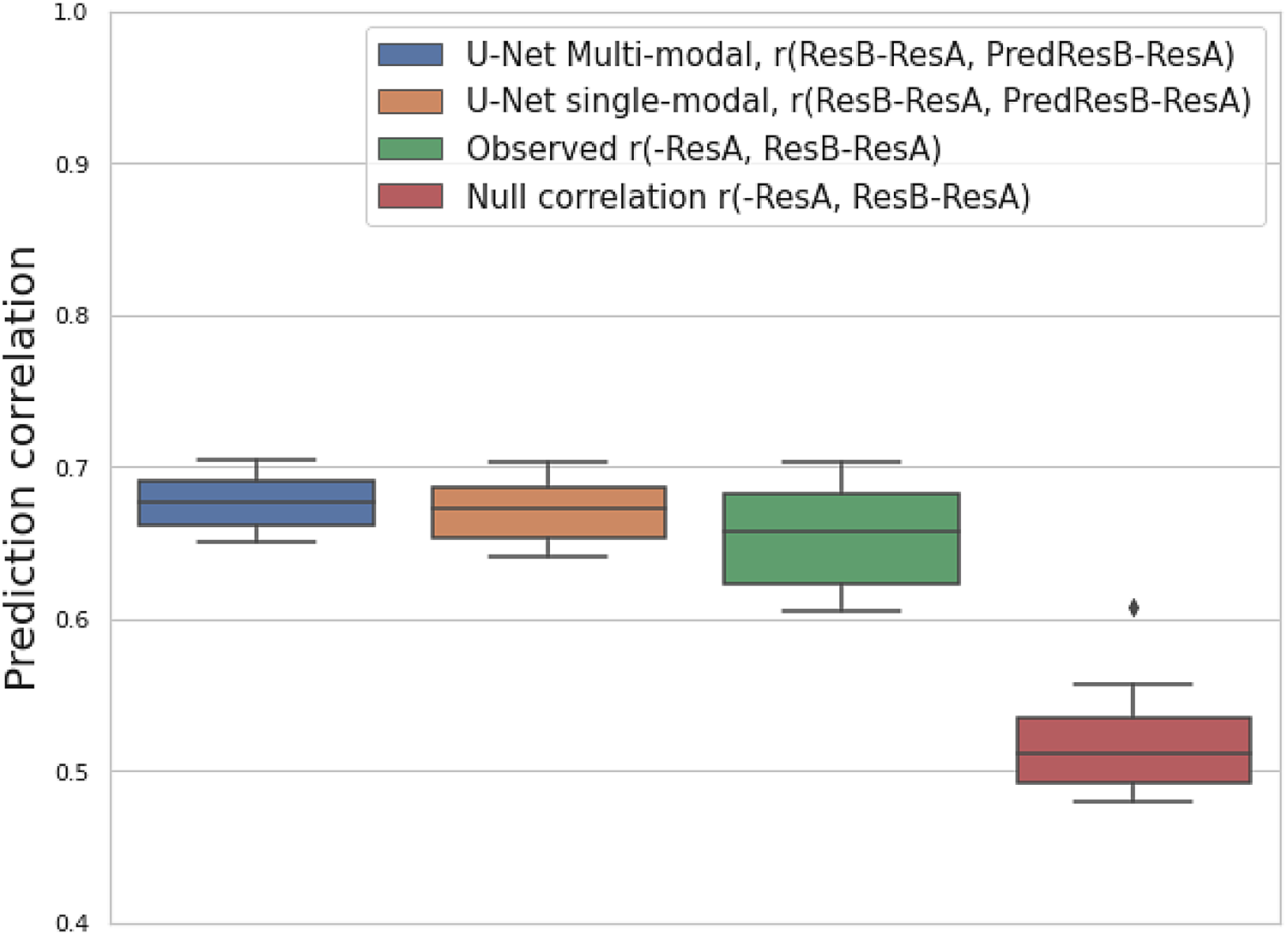
An analysis of model performance on predicting temporal difference images based on the split-half reproducibility of resting-state fMRI modalities. For 25 resting-state fMRI modalities, the boxplots show the prediction correlation of temporal difference images (ResB-ResA) with multimodal and single-modal U-Net (blue and orange); and observed correlation of baseline image (ResA) and temporal difference images (green); and estimated null correlations when there is no temporal change (red).

Figure 6 further shows, for 25 resting-state components, the distributions of the prediction correlation of the temporal difference image; the empirically observed correlation between baseline image and the real temporal difference image; and the estimated null correlation between baseline image and temporal difference image when the longitudinal difference is zero (section 2.1.1). The prediction correlation for each modality is shown in Supplementary Figure 4. As shown in section 2.1.2, we observed a higher correlation of the observed temporal difference than the null temporal difference, indicating a negative relation between the baseline image and the underlying longitudinal change. We observe that the prediction correlation of temporal difference is larger than the null correlation, but only slightly higher than copying -ResA as the prediction. The latter indicates that the main temporal change may negatively correlate with the baseline on its own, and the nonlinear parts of changes are small.

**Fig. 7.**
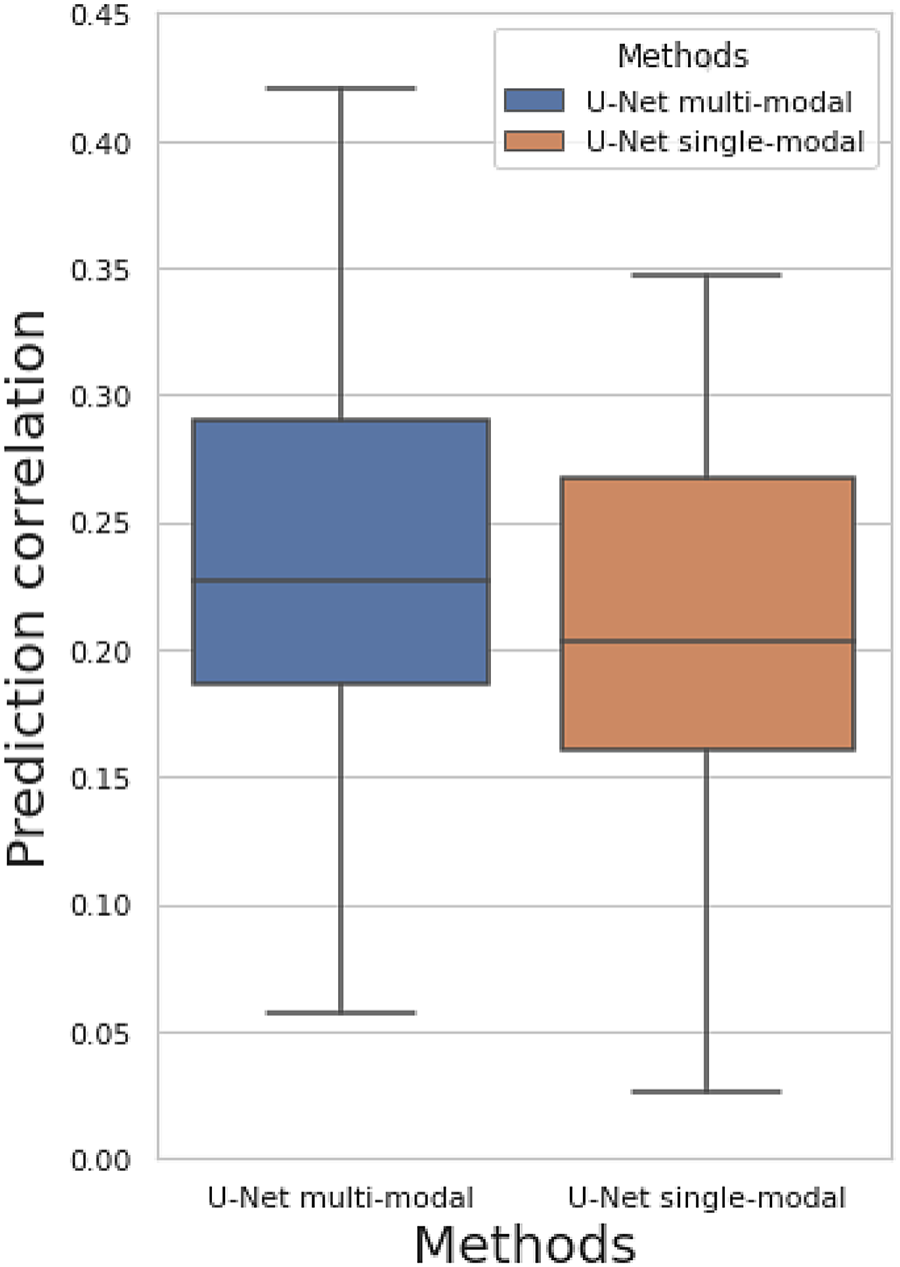
Prediction correlation of residual temporal difference image (ResB\ResA) among 50 brain imaging modalities.

Figure 7 shows the results for predicting residual temporal difference (ResB\ResA). Both multimodal and single-modal U-Net have prediction correlations clearly larger than zero. The baseline correlation here is zero because all linear information related to the baseline image has been removed. We observe lower overall predictiosn correlation values for predicting residual temporal difference images (ResB\ResA) than temporal difference images (ResB-ResA). This may be owing to the residualisation also removing linear relationships between baseline and longitudinal changes. In other words, we are trying to predict the nonlinear changes related to baseline images. The prediction correlation for each modality is shown in Supplementary Figure 5.

### 3.3 Test of whether a model performs change prediction or data denoising

We proposed to quantitatively test whether a trained model actually achieves longitudinal change prediction versus just denoising the data (section 2.1.3). Simply, we estimate ResB from ResA and compare this against predicting ResA from ResB. The former will be larger than the latter if the model is doing change prediction, while it will be similar if it is doing only data denoising.

**Fig. 8.**
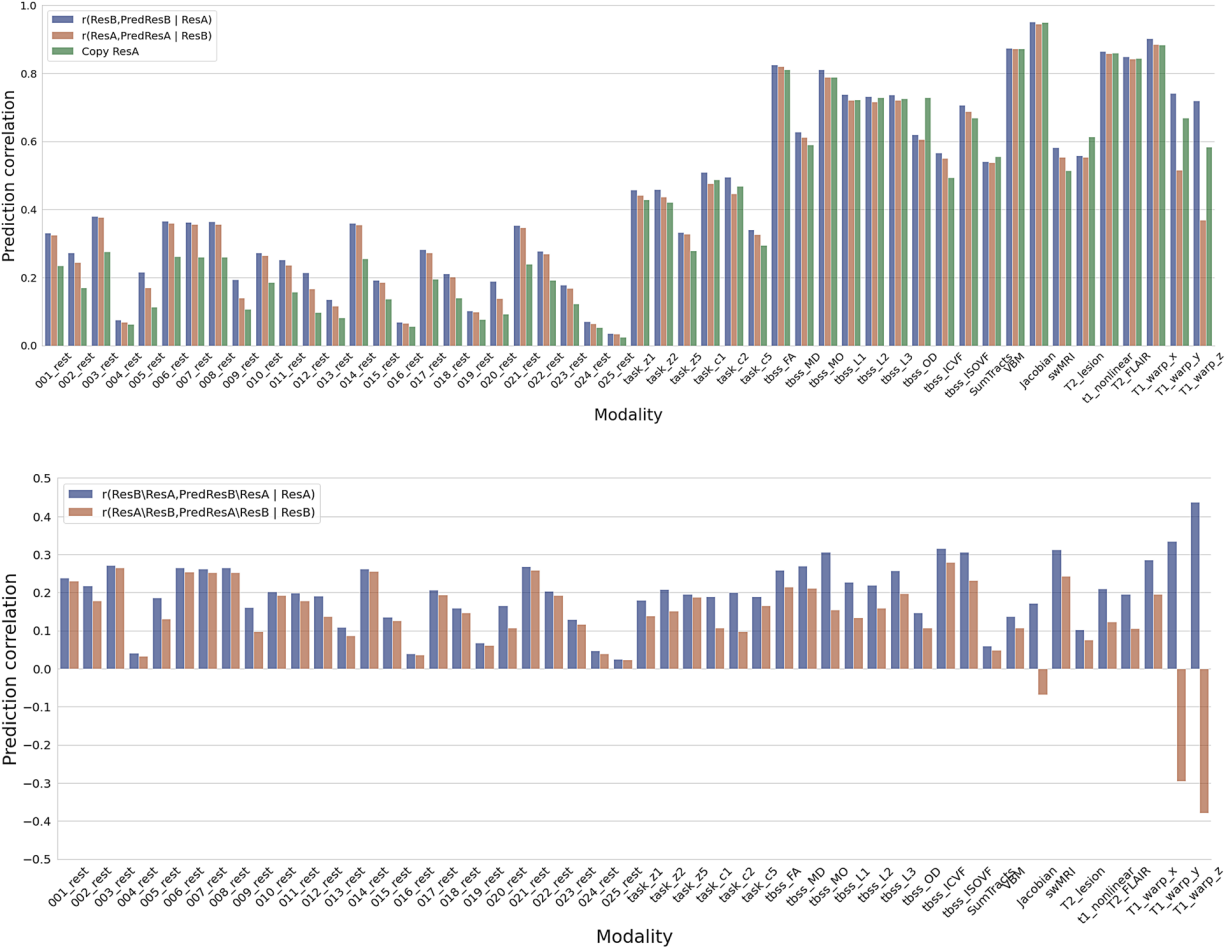
Prediction test to distinguish whether a model is carrying out longitudinal change prediction versus simple data denoising. Top: Given a trained model of predicting follow-up image (ResB) using baseline image (ResA), this compares the correlation of the output (PredResB) with the baseline image fed in (ResA) and the real follow-up image (ResB), that is, r(PredResB, ResB | ResA) (blue bars), against the correlation of output (PredResA) with the follow-up image (ResB) fed in and the real baseline image (ResA), that is, r(PredResA, ResA | ResB) (orange bars), and simple copy of baseline image (green bars). Bottom: Given a trained model of predicting residual temporal difference image (ResB\ResA) using baseline image (ResA), this compares the correlation of the output (PredResB\ResA) with the baseline image fed in (ResA) and the real residual temporal difference image (ResB\ResA), that is, r(PredResB\ResA, ResB\ResA | ResA) (blue bars), against the correlation of output (PredResA\ResB) with the follow-up image fed in (ResB) and the “inverse” real residual temporal difference image (ResA\ResB), that is, r(PredResA\ResB, ResA\ResB | ResB) (orange bars).

Figure 8 shows the results for analysing the prediction of follow-up images (top) and residual temporal difference images (bottom). For most modalities, the correlations of feeding baseline images into the trained network are larger than the correlation of feeding follow-up images into the network (and predicting “backwards”), suggesting a success of predicting longitudinal changes (Fig. 8, top). A similar conclusion holds for predicting the residual temporal difference images (Fig. 8, bottom). Specifically, these modalities follow other distinct patterns of results. For some rfMRI components (e.g., 1, 3, 6, 7, 8), A-predicts-B is a little better than B-predicts-A, and both are quite a lot better than A-equals-B (prediction is set equal to input); this suggests that for these modalities, there is a very small amount of successful longitudinal prediction, and a much larger amount of input denoising. For other modalities (e.g., rfMRI 5), the order of the 3 results is the same, but with more equal spacing, suggesting that the success of longitudinal prediction is more similar to the strength of the denoising. In some other modalities (e.g., rfMRI 4), all three analyses are similar and low, suggesting that almost no successful prediction or denoising is taking place. This example makes sense, as rfMRI 4 is one of the 4 rfMRI artefact components. Finally, in a few modalities (e.g., T1_warp_z), A-predicts-B is much higher than B-predicts-A, with A-equals-B in between; this suggests that longitudinal prediction is being highly successful, with little denoising taking place. In the analysis of predicting residual temporal difference images, we observed an interesting negative prediction correlation in Jacobian maps. The Jacobian deformation map is indicative of local volume changes, reflecting the longitudinal decrease in brain volume. Therefore, when inputting the follow up image into the model, it is expected to generate a map that shows the decreased volumes of the brain. Notably, this generated map exhibits a negative correlation with the “inverse” real residual temporal difference image (ResA\ResB). This finding demonstrated the capability of our model to adeptly capture and reflect the longitudinal patterns associated with brain volume reduction.

### 3.4 Discriminability of prediction

**Fig. 9.**
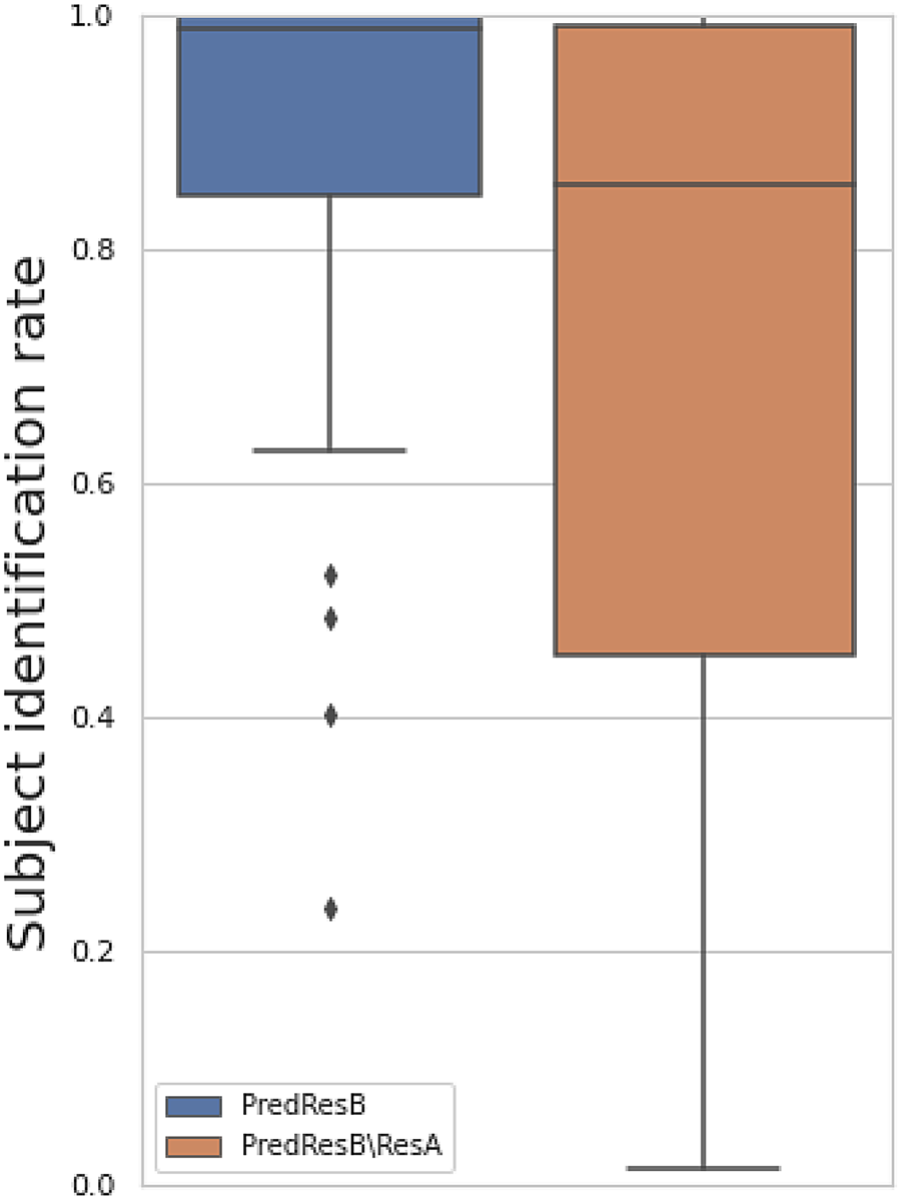
The subject identification rate of the predicted maps. We correlate each of the predicted maps to all the true maps. This shows the proportion of subjects whose within-subject correlation is the largest among all between-subject correlations.

A model can arguably achieve individualised prediction if the predicted spatial maps can be a subject’s fingerprint, that is, the predicted maps correlate most to the subjects themselves and are less correlated with other subjects. We tested whether the predicted maps could be identified based on their correlation with the true maps. Figure 9 shows that both the predicted follow-up images and the residual temporal difference images have a high discriminability approaching 1 (perfect subject identification rate) for many modalities, suggesting that our model can achieve individualised prediction. The high identification rate of PredResB may be driven by it simply looking like ResA, so we included residual temporal difference images in the same plot, which show that the PredResB\ResA are also “individualised”. PredResB\ResA has an overall lower discriminability than PredResB; this may be because the former has a worse prediction correlation so that less individual-level information is well captured. Finally, note that PredResB\ResA has a very low discriminability in some structure-related modalities, such as Jacobian maps and nonlinear warps. This may be because in these modalities the age-related structural changes are more common among subjects.

### 3.5 Relationships between predicted images and nIDPs

Our method has shown (in some cases) reasonable correlations between predicted and true images; we further investigated whether the predicted longitudinal changes relate to nIDPs. To this end, we used the *predicted residual temporal difference images* and the *true temporal difference image* to predict each of 17,485 nIDPs available in the UK Biobank. Supplementary Figure 6 shows the Bland-Altman plots comparing the prediction correlations of nIDPs. We see that in 34 of the 50 modalities, we have significantly improved the prediction performance of nIDPs with predicted maps compared with true maps. The improved prediction may result from a joint effect of U-Net longitudinal prediction and data denoising abilities. However, the predictions here only represent longitudinal associations between change in brain imaging and nIDPs, and the effect can be in both directions as we have included multiple variables measures before the brain imaging scans. We further selected 1,114 nIDPs from the 15,697 which measured at follow-up time points in the above analysis. Supplementary Figure 7 shows that the average prediction performance is very similar across different nIDPs and modalities (as shown by the difference in r), indicating the predicted images on average successfully captured the longitudinal patterns of nIDPs. Supplementary Table 1 shows the top 20 cognitive and health outcome variables that are best predicted by the predicted images and the true images.

However, importantly, when we explore the relationships between longitudinal changes and nIDPs, neither of the two definitions of longitudinal changes, that is, ResB-ResA or ResB\ResA, can perfectly remove the effect of baseline images. This is because ResB-ResA is explicitly correlated with the baseline image, and ResB\ResA can be correlated with the underlying true signals in ResA due to regression dilution problem (ResA contains both signal and noise, so that regressing out ResA does not necessarily means the true underlying signals have been fully regressed out).

Finally, for the 25 resting-state modalities, we explore the relationship of “mean improvements of nIDP predictions using the predicted maps vs. the true maps” with “the difference between prediction correlations of follow-up images and their test-retest reproducibility”. Supplementary Figure 8 shows that there is a clear linear relationship between these two metrics. This result indicates that the more accurately we can predict the follow-up images, compared with data reproducibility, the more we will improve with the predicted maps.

In summary, the above results show that our model can reasonably capture nIDP-related patterns in temporal changes in some cases, and may reduce the noise level of predicted data, resulting in similar or even improved nIDP prediction accuracy. Therefore, our prediction model can potentially impute imaging data of missing subjects in longitudinal studies and perform early prediction of brain imaging biomarkers.

### 3.6 Real data qualitative analysis

**Fig. 10.**
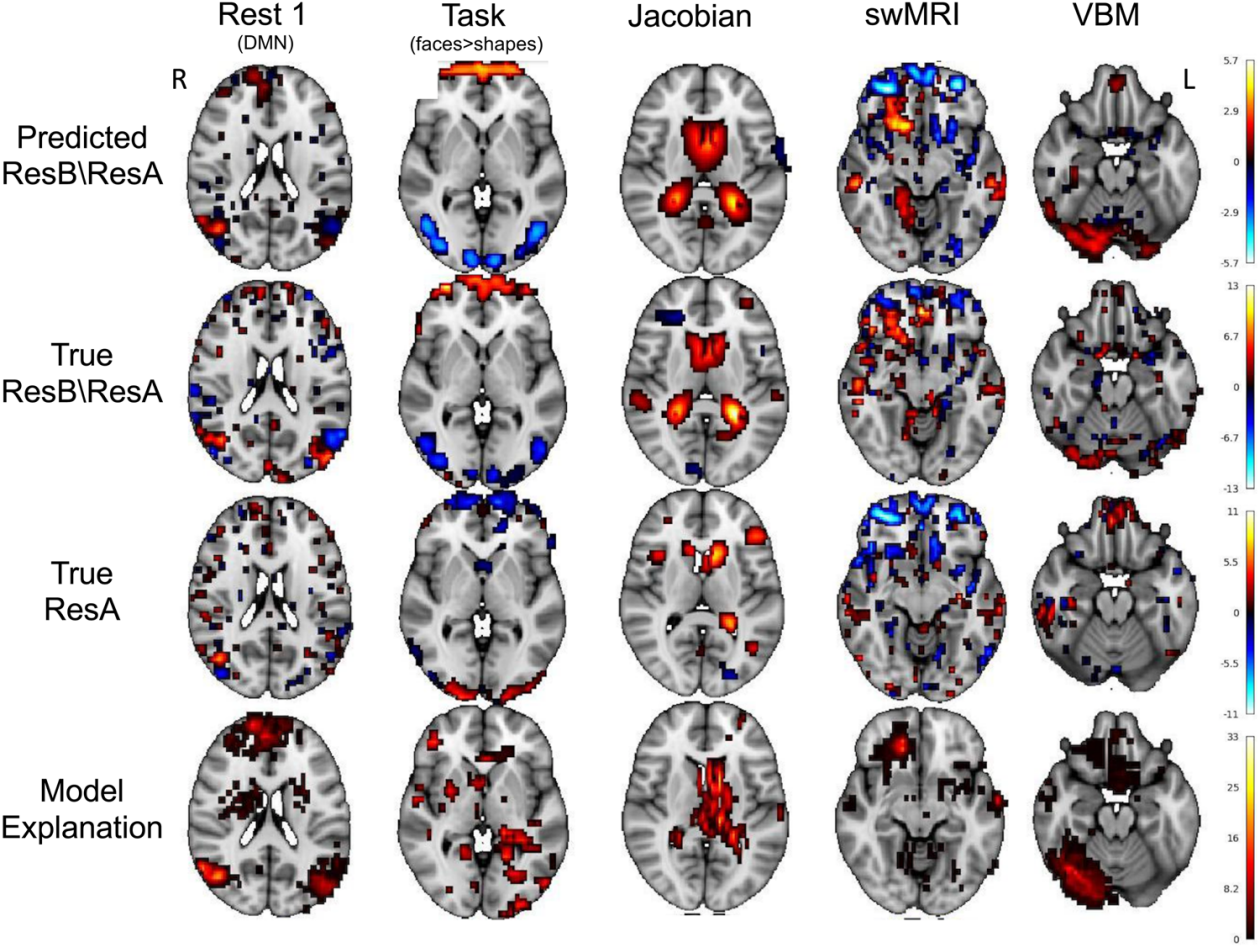
Single-subject examples of predicting residual temporal difference images and the prediction model explanations. Row 1: the predicted residual temporal difference images; Row 2: the true residual temporal difference images; Row 3: the true baseline image (input to the U-Net model); and Row 4: Single-subject explanation of model prediction (The parts of the brain in the baseline image that are predictive of residual temporal differences). The images are thresholded to show the voxels that have the highest 5% absolute values. In the model explanation, the voxels with higher absolute values are more important in prediction. Note that different columns are different subjects.

Figure 10 shows some examples of subjects’ predicted residual difference images (row 1) together with their true residual difference images (row 2), the input baseline image (row 3), and the model explanation of how the deep model predicts residual temporal difference using the baseline image (row 4). Each of them is thresholded only to include the top 5% of voxels based on the absolute values within the brain.

There are a lot of individual differences in different modalities that can be captured by the deep model. For example, in rest 1, that is, the resting-state default mode network, there are connectivity changes well predicted in the lateral occipital cortex, especially the positive-negative gradients in the left cortex. These changes are not reflected in the baseline image, but can be captured by the deep model as shown in the model explanation. Similarly, for the task contrast map, changes in task activations in the visual cortex are well predicted. For the T1 Jacobian determinant, an increase in the volume of the ventricles is well predicted by the model, which is a known ageing process. For swMRI, changes near the putamen and insular cortex are well predicted (likely reflecting iron deposition with ageing). For VBM, grey matter volume decreases in the occipital fusiform gyrus and lingual gyrus are well captured by the deep model.

**Fig. 11.**
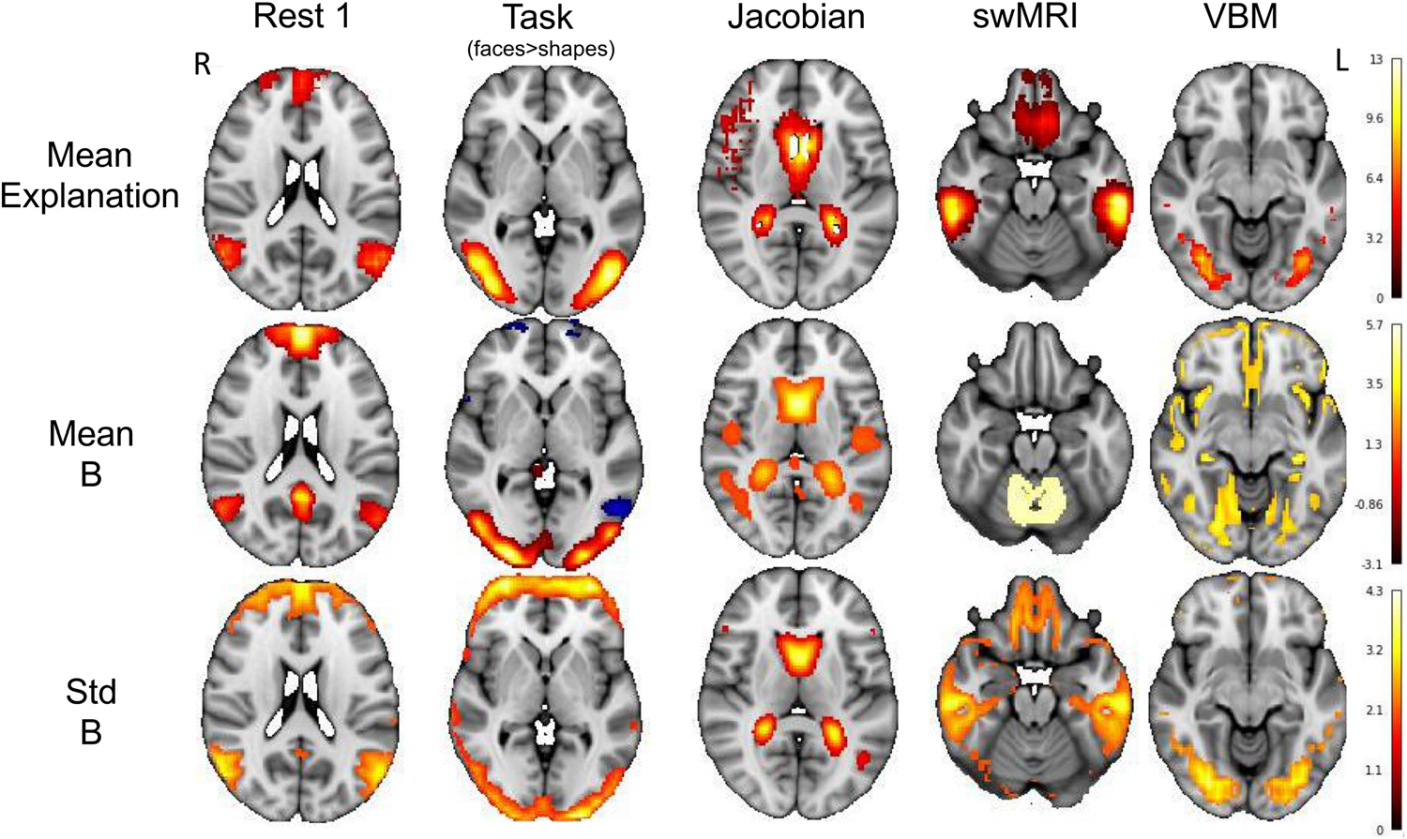
Group-averaged model explanation maps. Group mean of the parts of the brain in the baseline images that are predictive of residual temporal differences. Top: the population mean model explanation maps. The voxels with higher absolute values are more important in prediction; Middle: the population average maps of the follow-up images; Bottom: the population standard deviation of the follow-up images.

We further compared the group average model explanation of each modality (by averaging each individual’s explanation map across the populations) and the population average and standard deviation of follow-up images without residualisation. We show group average follow-up images without residualisation because the mean residualised maps are almost empty, as expected. Figure 11 shows some example results, corresponding to the modalities shown in Figure 10. We find that the deep model’s group average model explanation maps resemble (at least one of) the population average and population standard deviation of the follow-up images. The population mean and standard deviation maps reflect the parts of brains that have high values (e.g., high network connections, high task activations, and high volumes) and high between-individual variabilities. Note that the input of our deep model is the residualised baseline image, where the group information has been regressed out. However, our models are still able to learn to use the most informative part of the brain for prediction, partly reflecting that our model indeed captures individual variabilities in longitudinal change.

## 4 Discussion

In this paper, we proposed a general framework for individualised prediction of longitudinal changes in multimodal brain images. The proposed method takes one or multiple baseline brain imaging maps as inputs and outputs the predicted follow-up images and the temporal difference images. We show that we can predict the longitudinal changes to some degree in almost all of the 50 modalities in the UK Biobank, with 2-year follow-up time intervals. For example, this includes the brain shape changes reflected in the (alignment to standard space) warp fields, and the functional changes reflected in the resting-state network and task activation patterns. The method is generic and easy to train and applied to other modalities and datasets. We also proposed a mathematical framework for evaluating longitudinal predictions.

Compared with previous work (Da Silva et al., 2021; Gafuroğlu et al., 2018; Ghribi et al., 2021; Hong et al., 2019; Nebli et al., 2020; Rachmadi et al., 2020; Xia et al., 2021), our work here has the following advantages: First, for the first time, we proposed a mathematical modelling framework for longitudinal brain imaging studies. Based on this framework, we can (1) derive empirical “null” correlation of temporal change prediction; (2) demonstrate the existence of temporal changes in the imaging data; and (3) test whether a model achieves change prediction or denoises the data. These analyses are particularly important when the follow-up time interval is small (e.g., around 2 years in the UK Biobank data). Second, our method is more generic, and the same U-Net structure can be applied to train for 50 different modalities in one of the largest longitudinal neuroimaging datasets UK Biobank. The sample size and number of modalities used here are significantly larger than in previous studies. Third, the proposed method is further validated by comparing the predicted maps with the true maps in terms of predicting thousands of nIDPs in the UK Biobank. Finally, we also showed that the predicted follow-up maps and longitudinal changes could be fingerprints identifying a subject.

Our paper points out various caveats of longitudinal prediction. It is important to have a meaningful definition of longitudinal changes. We proposed the *temporal difference* and the *residual temporal difference* as two definitions. However, both have their pros and cons. The temporal change is the most straightforward one, but we need to carefully derive its “null” prediction correlation, because it intrinsically contains a baseline image term. The residual temporal difference partially overcomes this problem, but may overly regress out temporal changes that are linearly related to the baseline images, and may also suffer from the regression dilution problem so that the (true underlying) signals in the baseline image are still there. However, due to the high noise in both baseline and follow-up neuroimaging data, a “perfect” definition that can fully remove baseline image information from longitudinal change is hard to find. Therefore, when interpreting longitudinal results, we need carefully think about whether the findings are truly related to the changes or just confounding factors.

We could further improve the current work in the following ways. First, the prediction performance of follow-up images or temporal difference images may be improved if we incorporate nIDP information as conditions in the training process. Second, U-Net based prediction models are known to easily capture low-frequency signals and sometimes unable to learn the high-frequency details (Schwarz et al., 2021; Y. Wang et al., 2021). Designing more advanced architectures to capture details in high-resolution brain images may improve longitudinal prediction. Third, for all models, longitudinal prediction and image denoising are always mixed together in longitudinal prediction task. A mathematical framework which explicitly models and distinguishes the two effects may be beneficial for future work to quantitatively estimate the success of longitudinal prediction.

In summary, our study contributes a new theoretical framework for longitudinal brain imaging studies, and our results show the potential for longitudinal data imputation, along with highlighting several caveats when performing longitudinal data analysis. The framework may be beneficial for longitudinal brain imaging-based neuroscientific studies.

## Supplementary Material

Supplementary Material

## Data Availability

UK Biobank data are available from UK Biobank via their standard data access procedure (see http://www.ukbiobank.ac.uk/register-apply). The code for longitudinal prediction model is released on **Github**: https://github.com/weikanggong/LongitudinalPrediction.
