# Supplementary material for: Individualised prediction of longitudinal change in multimodal brain imaging
Source: Imaging Neurosci (Camb). 2024 Jul 3;2:imag-2-00215. doi: 10.1162/imag_a_00215 (PMC11877422; doi:10.1162/imag_a_00215)
Supplement: Supplementary Material [file imag_a_00215-supp.pdf]

# Supplemental material of Individualised Prediction of Longitudinal Change in Multimodal Brain Imaging

Weikang Gong,[1,2*] Christian F. Beckmann,[2,3,4] Stephen M. Smith[2]

[1]School of Data Science, Fudan University, Shanghai, 200433, China.

[2]Centre for Functional MRI of the Brain (FMRIB), Nuffield Department of Clinical Neurosciences, Wellcome Centre for Integrative Neuroimaging, University of Oxford, Oxford, UK.

[3]Radboud University Medical Centre, Department of Cognitive Neuroscience, Nijmegen, Netherlands.

[4]Donders Institute for Brain, Cognition and Behaviour, Radboud University Nijmegen, Nijmegen, Netherlands. *Correspondence: weikang.gong@ndcn.ox.ac.uk

## 1 An example of estimating the noise level of brain images.

For example, for resting-state fMRI dual-regression spatial maps, we can estimate the $\sigma$ based on split-half reproducibility. That is, for each subject, we split the whole time series into two equal-length parts using baseline data, re-estimate the subject-level dual-regression spatial maps (using the same group-level ICA maps), and then compute the correlation between the two estimated maps. We name this correlation "reproducibility". Note that in this case, the estimated reproducibility is smaller than the true reproducibility, because we only use half of the time series. Denoting the noise variance with split-half data as $\sigma^2_{\text{half}}$, we have:

$$\begin{aligned} A_1 &= A_0 + E_{A_1} \\ A_2 &= A_0 + E_{A_2} \\ r(A_1, A_2) &= \frac{1}{1+\sigma^2_{\text{half}}} \end{aligned} \tag{1}$$

And from the equations for estimating dual-regression spatial maps, we can derive the relationship between the true $\sigma^2$ and $\sigma^2_{\text{half}}$ as:

$$\sigma^2 = \sigma^2_{\text{half}}/2 \tag{2}$$

For example, as shown later in the red bars in Fig. ??, an estimate of split-half reproducibility for resting-state dual regression spatial map 1 (i.e., the default mode network) is 0.34; taking this number into the above equations, we can get an estimate of $\sigma^2 = 0.97$. Taking this estimated $\sigma^2$ back into Eq. ?? ($\Delta = 0$ case), we get the "null" correlation $r(B-A,-A) = 0.49$. This means that in the null case of no temporal changes existing, simply copying $-A$ as the prediction of $B-A$ can have a high correlation of 0.49.

# 2 Supplementary Figures

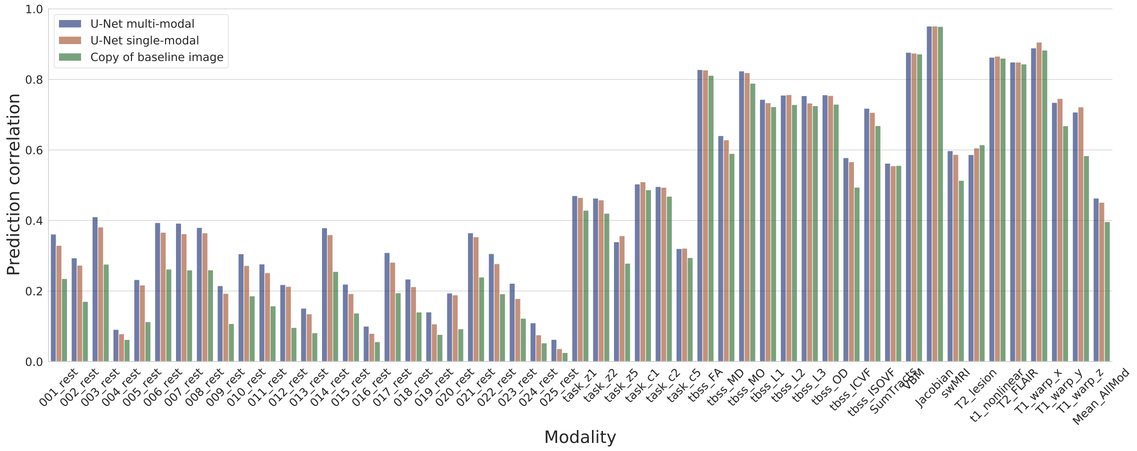


**Figure 1: Prediction correlation of follow-up image with the baseline image using the proposed model**. Prediction correlation of follow-up images (ResB) with multimodal and single modal U-Net, and a baseline of copying baseline image (ResA) as the prediction. The mean correlations across all modalities are shown on the right.

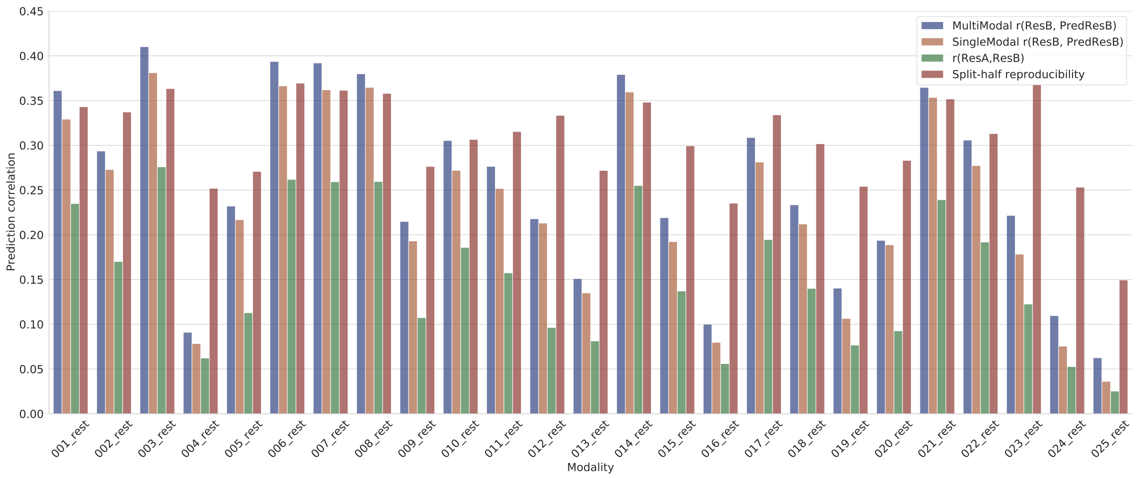


**Figure 2: Evaluation of model performance on predicting follow-up images based on the split-half reproducibility of resting-state fMRI modalities**. For 25 resting-state fMRI modalities, the prediction correlation of follow-up images (i.e., ResB) with multimodal and single modal U-Net, and a baseline of copying baseline image (i.e., ResA) as the prediction, and the test-retest reproducibility by splitting the time series in half. The mean correlations across all modalities are shown on the right. Among the 25 modalities, component 4, 23, 24, 25 have been identified as non-neural components.

**Table 1:** Top 20 cognition and health outcome nIDPs that are best predicted by the residual temporal difference images.

| nIDPs that best predicted by *real* longitudinal changes | | | |
|---|---|---|---|
| nIDPs | r(predicted images) | r(real images) | modality |
| Leg pain on walking : effect of standing still | 0.026 | 0.208 | task_z2 |
| Worrier / anxious feelings | 0.085 | 0.208 | task_c5 |
| FI7 : synonym | 0.030 | 0.198 | task_c2 |
| Fluid intelligence score | 0.002 | 0.193 | task_c1 |
| Leg pain on walking : effect of standing still | 0.038 | 0.186 | task_z1 |
| Mineral and other dietary supplements | 0.063 | 0.185 | task_z1 |
| FI9 : concept interpolation | 0.009 | 0.173 | task_c5 |
| FI7 : synonym | 0.032 | 0.171 | task_c1 |
| Guilty feelings | 0.045 | 0.165 | task_c5 |
| Frequency of tiredness / lethargy in last 2 weeks | 0.076 | 0.164 | task_z5 |
| Fed-up feelings | 0.092 | 0.160 | task_c5 |
| Fluid intelligence score | 0.024 | 0.158 | task_c2 |
| Neck/shoulder pain for 3+ months | 0.000 | 0.157 | task_c2 |
| Number of word pairs correctly associated | 0.071 | 0.153 | task_c2 |
| Duration to entering selection | 0.042 | 0.153 | task_c1 |
| Worrier / anxious feelings | 0.079 | 0.152 | task_z5 |
| Knee pain for 3+ months | 0.034 | 0.151 | task_z2 |
| Guilty feelings | 0.143 | 0.150 | task_c2 |
| FI6 : conditional arithmetic | 0.075 | 0.147 | task_z1 |
| Age diabetes diagnosed | 0.079 | 0.145 | rest 8 |
| **nIDPs that best predicted by *predicted* longitudinal changes** | | | |
| Taking other prescription medications | 0.195 | 0.052 | task_z1 |
| Interval between previous point and current one in numeric path (trail #1) | 0.190 | 0.000 | task_z2 |
| Friendships satisfaction | 0.177 | 0.078 | task_c5 |
| Hearing difficulty/problems | 0.176 | 0.034 | task_c2 |
| Long-standing illness, disability or infirmity | 0.173 | 0.021 | task_z2 |
| Non-cancer illness year/age first occurred | 0.164 | 0.088 | task_c1 |
| Interval between previous point and current one in alphanumeric path (trail #2) | 0.161 | 0.111 | task_z1 |
| Years since last breast cancer screening / mammogram | 0.156 | 0.000 | task_z2 |
| Time first key touched | 0.153 | 0.053 | task_z2 |
| Value entered | 0.150 | 0.052 | task_c2 |
| Work/job satisfaction | 0.148 | 0.129 | task_c2 |
| Interval between previous point and current one in alphanumeric path (trail #2) | 0.147 | 0.091 | task_z5 |
| Duration to entering selection | 0.144 | 0.098 | task_z1 |
| Ever highly irritable/argumentative for 2 days | 0.143 | 0.087 | task_c5 |
| Guilty feelings | 0.143 | 0.150 | task_c2 |
| Time last key touched | 0.143 | 0.000 | task_z2 |
| Duration to entering symbol choice | 0.142 | 0.096 | task_z5 |
| Fluid intelligence score | 0.142 | -0.027 | task_c5 |
| Value entered | 0.141 | 0.025 | task_z2 |
| Value entered | 0.140 | 0.015 | task_z1 |
| Duration to complete numeric path (trail #1) | 0.140 | 0.062 | task_c1 |
| Back pain for 3+ months | 0.139 | 0.074 | task_c1 |

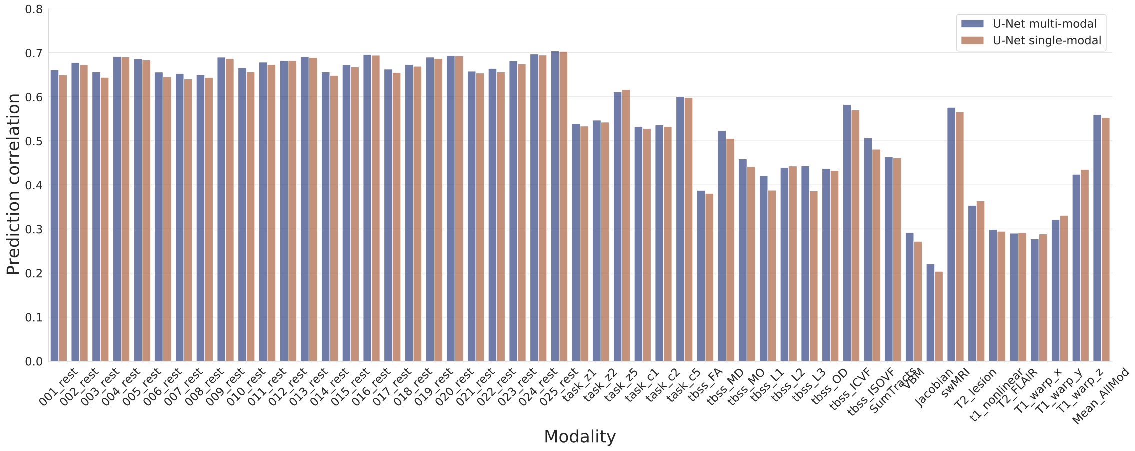


**Figure 3: Prediction performance of temporal difference images**. Prediction correlation of temporal difference images (ResB-ResA) with multimodal and single modal U-Net. The mean correlations across all modalities are shown on the right.

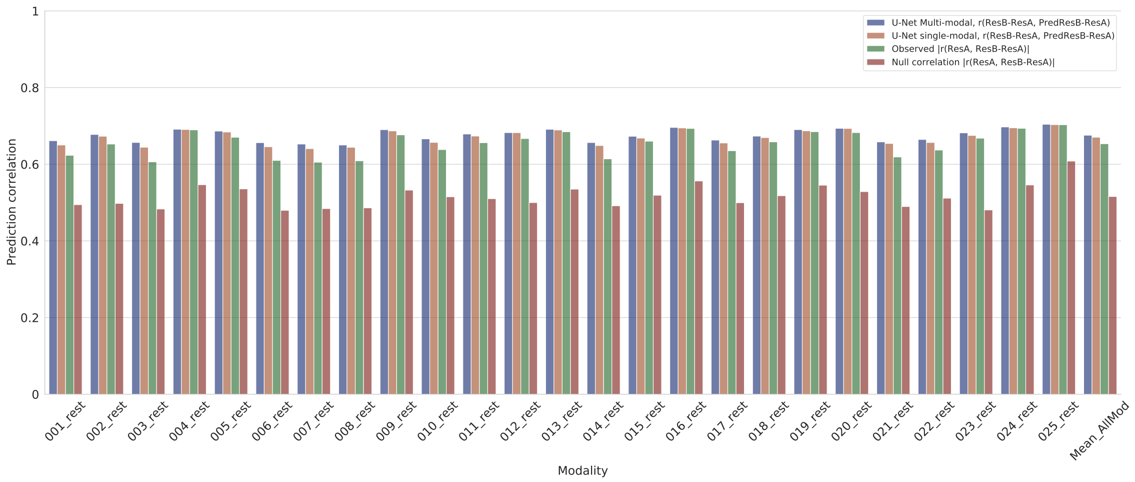


**Figure 4: Evaluation of model performance on predicting temporal difference images based on the split-half reproducibility of resting-state fMRI modalities**. For each of the 25 resting-state modalities, the 4 bars shows (1) the prediction correlation of temporal difference images (ResB-ResA) with (1) multimodal and (2) single modal U-Net; (3) a baseline of copying the baseline image (-ResA) as the prediction; (4) the estimated null correlation when there are no longitudinal changes. The mean correlations across all modalities are shown on the right.

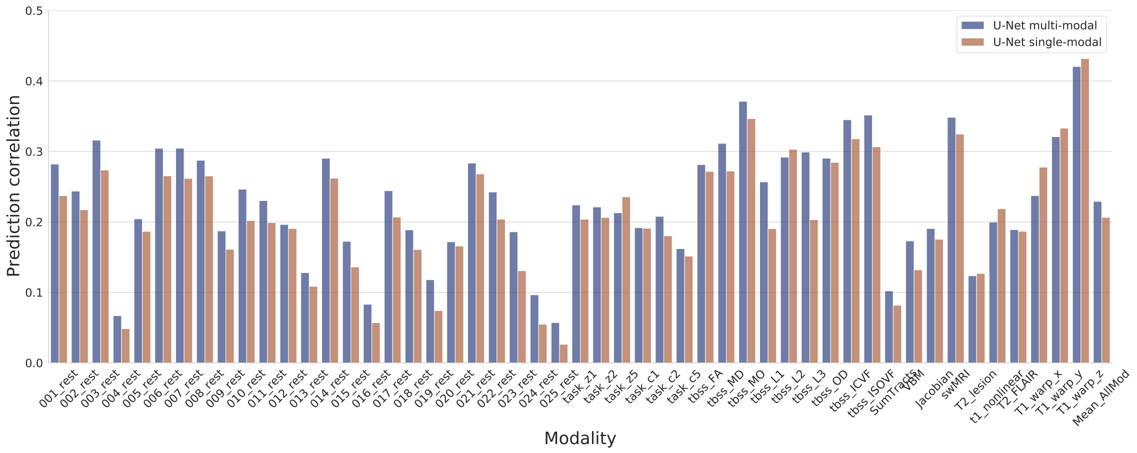


**Figure 5: Prediction performance of residual temporal difference images**. Prediction correlation of residual temporal difference images (ResB\ResA) with multimodal and single modal U-Net. Here the baseline correlation is zero because ResA has been regressed out. The mean correlations across all modalities are shown on the right.

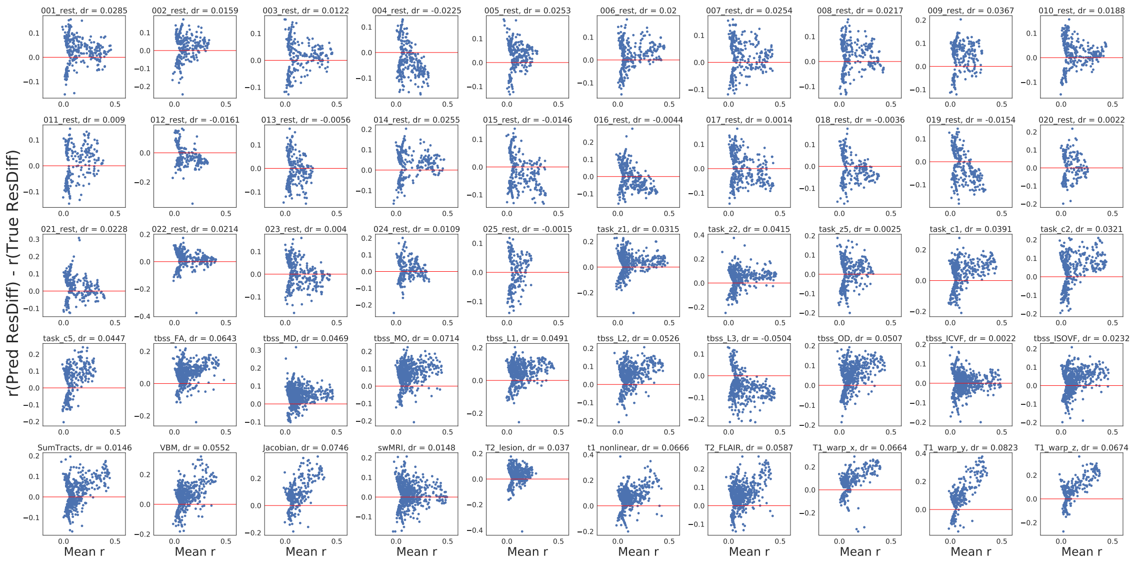


**Figure 6: Bland-Altman plots of nIDP prediction performance using the predicted and true residual temporal difference images.** Each point in the figure represents one of the 15,697 nIDPs, where the x-axis is the mean of its prediction correlation and the y-axis is the difference of the correlation between predicted images and true images.

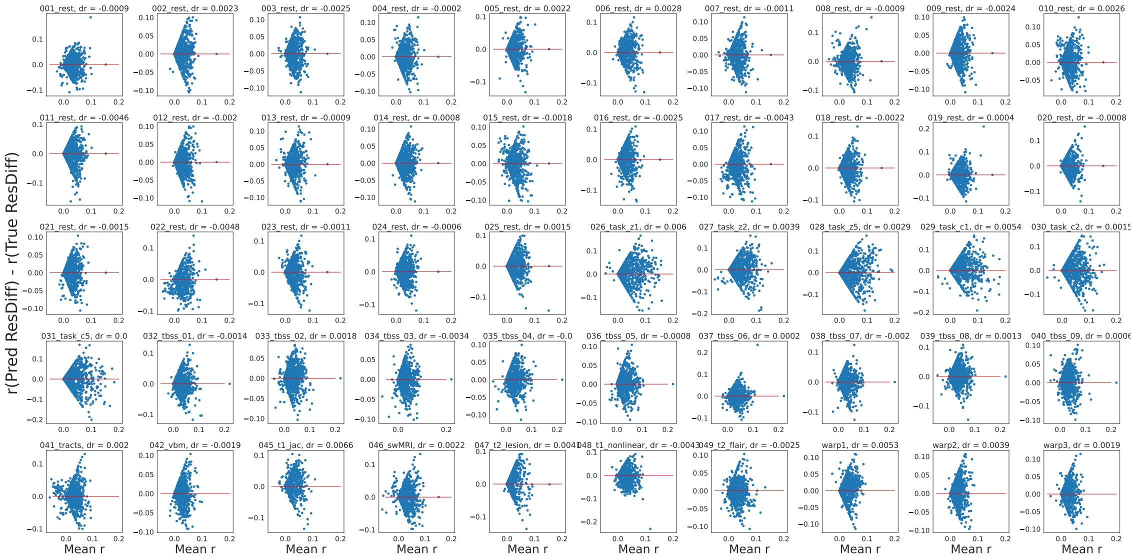


**Figure 7: Bland-Altman plots of nIDP prediction performance using the predicted and true residual temporal difference images.** Each point in the figure represents one of the 1,114 nIDPs measured at follow-up brain imaging scan, where the x-axis is the mean of its prediction correlation and the y-axis is the difference of the correlation between predicted images and true images.

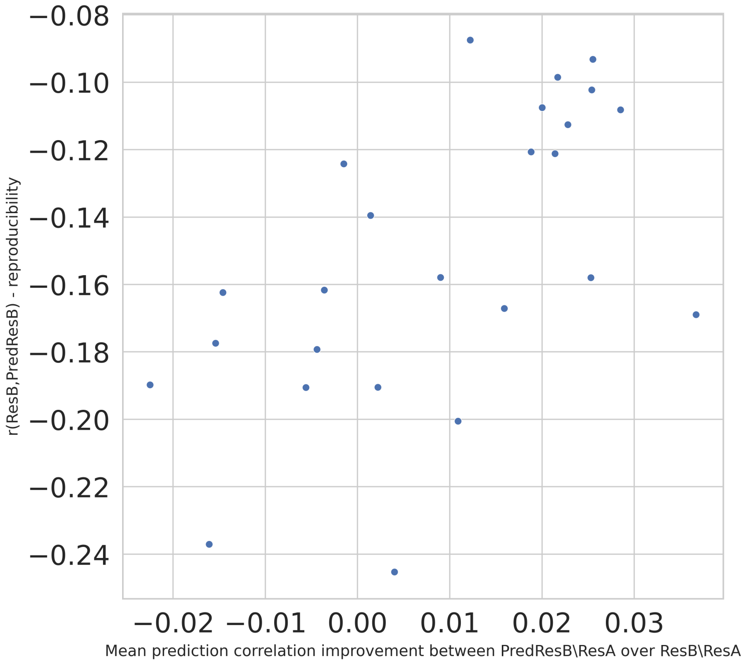


**Figure 8:** The improvements of prediction correlation of nIDPs between predicted and true residual temporal difference images positively correlated with the difference of follow-up image prediction correlation and test-retest reproducibility ($r = 0.59, p = 0.002$) in 25 resting-state fMRI modalities.
